# Induction of an immortalized songbird cell line allows for gene characterization and knockout by CRISPR-Cas9

**DOI:** 10.1038/s41598-022-07434-7

**Published:** 2022-03-14

**Authors:** Matthew T. Biegler, Olivier Fedrigo, Paul Collier, Jacquelyn Mountcastle, Bettina Haase, Hagen U. Tilgner, Erich D. Jarvis

**Affiliations:** 1 Laboratory of Neurogenetics of Language, The Rockefeller University, 1230 York Avenue, New York, NY 10065 USA; 2 Howard Hughes Medical Institute, Chevy Chase, MD USA; 3 Vertebrate Genome Laboratory, The Rockefeller University, New York, NY 10065 USA; 4 Center for Neurogenetics, Graduate School of Medical Sciences, Weil Cornell Medical Center, New York, NY 10065 USA

**Keywords:** Biological techniques, Cell biology, Neuroscience

## Abstract

The zebra finch is one of the most commonly studied songbirds in biology, particularly in genomics, neuroscience and vocal communication. However, this species lacks a robust cell line for molecular biology research and reagent optimization. We generated a cell line, designated CFS414, from zebra finch embryonic fibroblasts using the SV40 large and small T antigens. This cell line demonstrates an improvement over previous songbird cell lines through continuous and density-independent growth, allowing for indefinite culture and monoclonal line derivation. Cytogenetic, genomic, and transcriptomic profiling established the provenance of this cell line and identified the expression of genes relevant to ongoing songbird research. Using this cell line, we disrupted endogenous gene sequences using *S.aureus* Cas9 and confirmed a stress-dependent localization response of a song system specialized gene, SAP30L. The utility of CFS414 cells enhances the comprehensive molecular potential of the zebra finch and validates cell immortalization strategies in a songbird species.

## Introduction

Songbirds are extensively utilized to study vocal learning and communication^1,2^, sexual dimorphism^3^, comparative evolution^4,5^, and more. The zebra finch (*Taeniopygia guttata castanotis*) is especially useful for these diverse fields as it breeds easily in captivity^6^. Thus, more genomic resources^7,8^ and neuroscience tools^9–12^ have been developed for the zebra finch than for any other songbird species^6^. However, cell culture tools remain underdeveloped.

Cell culture is a valuable tool to evaluate reagents, express recombinant genes, and characterize cellular and molecular processes in a controlled and quantifiable manner. Previously, zebra finch cell lines have been established using an induced pluripotent stem cell (iPSC) construct, STEMCCA, containing four transcription factors^13^, or from tumors (G266 and ZFTMA) from a male and female, respectively^14^. However, these lines are difficult to maintain as culture stocks, compared to stable cell lines from other species, and have not been widely utilized across the avian science community. Primary cells have also been utilized^15,16^, though these cells have a limited lifetime in vitro. Primary cells also vary genetically between individuals and laboratories, risking issues with replicability between divergent zebra finch lab populations^17^. Particularly, none of these options allow for density-independent growth, which is useful for maintaining cell population homogeneity and generating transgenic cell lines. The lack of a robust, stable cell line hinders the use of molecular tools in the zebra finch, such as CRISPR-Cas9^10,11^. An immortalized cell line would allow for the exploration of the basic molecular and cellular understanding of this vocal learning species. Resolving the role of key genes involved in vocal learning pathways is challenging without efficient cell lines to test their function and optimize reagents for in vivo experiments.

In poultry (*Galloanserans* e.g., chickens and ducks), cell lines have been successfully established. The DF-1 chicken cell line was derived from a spontaneously transformed culture population^20^, while the DT40 chicken cell line was derived from a chicken infected with Rous sarcoma virus^21^. Both are critical for scientific and biotechnology applications^22,23^. Other avian cell lines include a duck embryonic fibroblast line, DEF-TA, that was immortalized with the Simian vacuolating virus 40 (SV40) large T antigen^24^. Importantly, each of these cells are capable of robust, density-independent survival and proliferation, enabling clonal propagation and antibiotic selection strategies. Such approaches have not been previously reported in passerine birds despite constituting over half (~ 5000) of all bird species^25^, representing a major gap in the toolset for studying this clade’s cellular and molecular biology. As we learn more about both the convergent and unique biological properties of songbirds^16,26,27^, the need for immortalized cell lines becomes increasingly clear.

Here, we established the provenance of an induced, continuous cell line from zebra finch embryonic fibroblasts using a lentivirus containing SV40 large and small T antigens (SV40Tt), capable of density-independent proliferation and clonal propagation. We characterized the genomic integrity and transcriptional landscape using next generation sequencing approaches, showing a relatively stable and multipotent cell line of myogenic origin that possessed a gene expression profile relevant to several songbird research areas. We further demonstrated the cell line’s applicability to test guide RNAs (gRNAs) for CRISPR-mediated genome editing and study genes related to vocal learning and other songbird traits.

## Results

### Establishment of a continuous cell line derived from zebra finch

**Figure 1:**
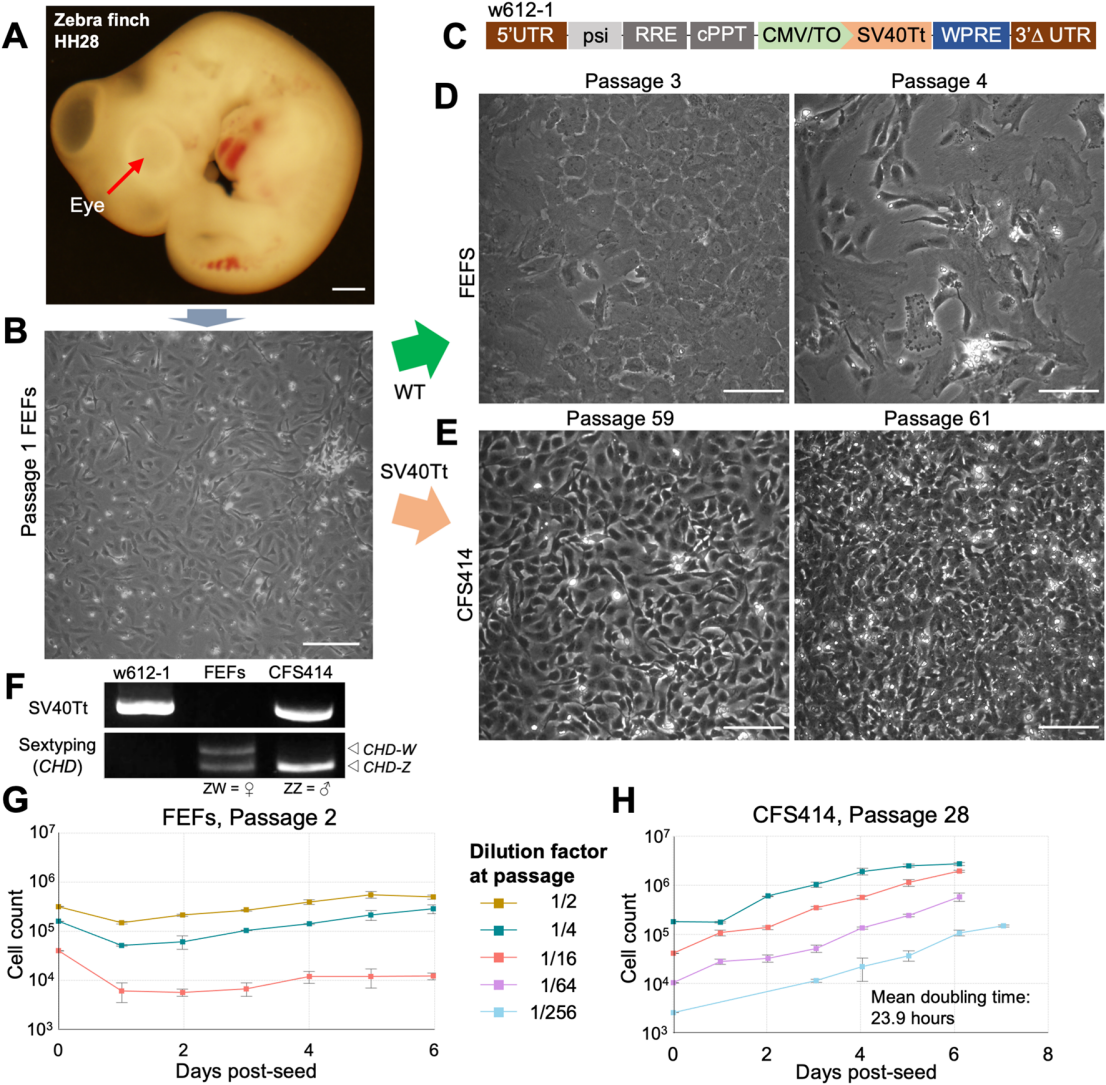
Generation of immortalized finch cells with SV40Tt. (**A**) Embryonic day 6 zebra finch with the CFW allele, imaged at 4 × magnification. Note the lack of eye pigmentation (red arrow). Scale bar = 200 µm. (**B**) Exemplar image of passage 1 finch embryonic fibroblast (FEF) culture. (**C**) Diagram of lentiviral construct (w612-1) delivering SV40Tt immortalization factor into the zebra finch genome. Abbreviations: LTR 5′ and 3∆′, Long terminal repeat sites; psi, retroviral packaging element; RRE, Rev responsive element; cPPT, central polypurine tract; CMV/TO, Cytomegalovirus promoter with 2 Tet responsive element motifs, allowing for inducible expression in the presence of Tet-repressor constructs; SV40Tt, Simian virus 40 large and small T antigen; WPRE, woodchuck hepatitis virus posttranslational responsive element. (**D**) Passage 3 FEFs at full confluence (left) and passage 4 from a 1/2 split (right), with low confluence 11 days after seeding. (**E**) CFS414 cells between passage 59 at full confluence (left) and passage 61 from a 1/10 split (right), overconfluent 5 days after seeding. Expanded images of passage 59 emphasize morphological diversity in Supplementary Fig. 1D. (**F**) PCR amplicons of SV40Tt and sex-chromosome specific *CHD* gene sequence (ZZ is male, ZW is female), using w612-1 and genomic DNA derived from primary female FEFs or the male CFS414 cell line (right). (**G, H**) Growth curves of (**G**) primary FEFs at passage 2 and (**H**) CFS414 cells at passage 28. Seeding densities range from 320,000 cells/well (~ 1/2 dilution) and 1250 cells/well (~ 1/256 dilution). Error bars denote SEM.

Building on studies of induced avian cell lines in poultry^21,24^, we sought to generate an immortalized cell line from the zebra finch. We dissociated skin and muscle tissue from Hamburger-Hamilton stage 28 (HH28) embryos (Fig. 1A) to derive finch embryonic fibroblast cultures (FEFs; Fig. 1B). These cells were left untreated or transduced with lentivirus expressing either the SV40 large and small T antigen (SV40Tt, Fig. 1C) or control GFP (Supplementary Fig. 1A). Compared to wild-type (Fig. 1D) and GFP-transduced FEFs (Supplementary Fig. 1B,C), SV40Tt-transduced cells proliferated robustly beyond several passages (Fig. 1E). The most proliferative SV40Tt-transduced cell line, hereafter named CFS414, was derived from a single chestnut-flanked white (CFW) embryo lacking eye pigmentation (Fig. 1A). This line contained several cell morphologies (Supplementary Fig. 1D). The presence of SV40Tt in CFS414 genomic DNA was confirmed with PCR (Fig. 1F). PCR sextyping using the *CHD* gene showed the presence of only the Z chromosome (Fig. 1F), indicating the cell line originated from a male embryo.

Compared to the limited growth potential of wild-type FEF controls at passage 2 over 6 days (Fig. 1G), CFS414 cells assessed at passage 28 were highly proliferative (Fig. 1H). The CFS414 cells also grew independently of initial seed density, maintaining an average doubling time of 23.9 h during log-phase growth (Fig. 1H), similar to other cell lines such as HEK293T cells (doubling time ~ 24–30 h; RRID: CVCL_0063). Even after 55 passages, the growth curve and doubling time remained stable (Supplementary Fig. 1E; p = 0.553, two-tailed Student’s t-test), suggesting low population-wide senescence. At high confluence, CFS414 cells continued growing with limited contact inhibition, and were resistant in forming "cell sheets," unlike wildtype FEFs (Supplementary Fig. 1F,G).

### Assessment of chromosome and genome sequence integrity

**Figure 2:**
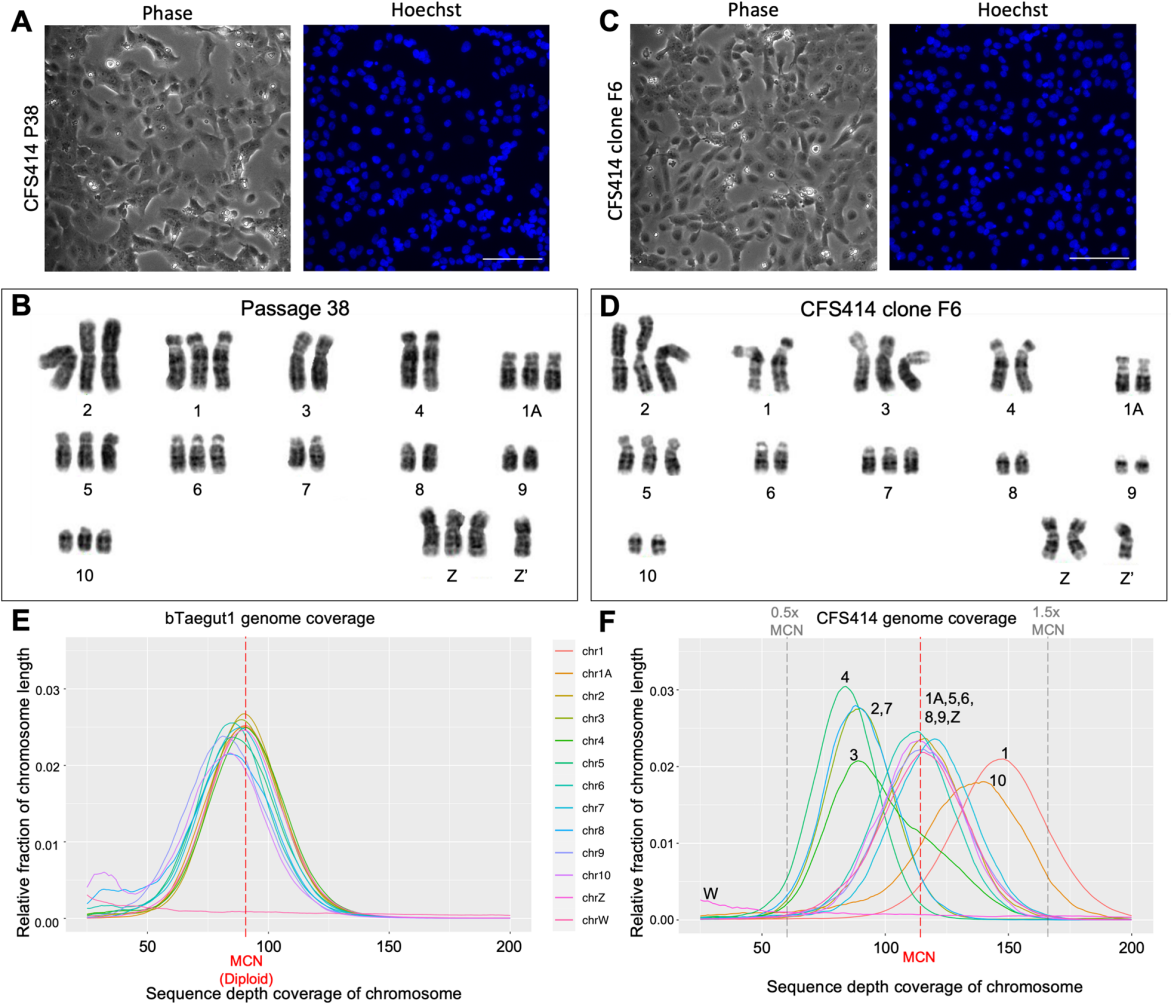
CFS414 chromosome copy numbers are variable. (**A, C**) Phase contrast images and accompanying Hoechst 33,342 nuclear stain images of (**A**) passage 38 and (**C**) CFS414 clone F6 cells derived by flow cytometry. (**B, D**) Corresponding Giemsa-stained macrochromosomes for (**B**) Passage 38 and (**D**) F6 cells. Chromosomes are arranged and numbered by size, with genome assembly chromosome numbers listed underneath each pair as identified through linkage-mapping from previous studies^31,32^. Trisomies are present for many chromosomes, with variability across cells. Note presence of mutant Z chromosome (Z′) in all karyotypes. See Supplementary Fig. 2 for further karyotype spreads. (**E**) Coverage peaks from the bTaegut1 male zebra finch genome, showing a single grouping of chromosomes at the MCN (the red dotted line; coverage 88.2). (**F**) Relative coverage for each chromosome in CFS414 cells. Distributed peaks suggest cell population-wide copy number differences b etween chromosomes. The flanking grey lines representing 0.5 × and 1.5 × the MCN for reference. Note the CFS414 MCN is not necessarily representative of diploid chromosome pairs. See Supplementary Fig. 3 for individual chromosome coverage plots.

Chromosomal abnormalities are common among cell lines^29^, particularly those transformed by SV40 T antigens^30^, and may impact the generalizable nature of cell lines to species biology. At passage 38 (Fig. 2A), G-banded macrochromosome karyotypes appeared normal and intact when compared to previous studies^13,15,31^, but trisomies in many chromosome pairs were common (Fig. 2B). Example cell spreads within the sampled populations were variable (e.g. Supplementary Fig. 2A,B). At passage 63 (Supplementary Fig. 2C), tetrasomies and single-copy chromosomes were identified (Supplementary Fig. 2D). A monoclonal line derived through flow cytometry (denoted clone F6, Fig. 2C) also had dissimilar karyotype spreads at only 7 passages after isolation (Fig. 2D; Supplementary Fig. 2E,F), suggesting the high nondisjunction rates were not simply related to high passage numbers or polyclonal populations in the cell line. In all spreads examined, multiple copies of the Z chromosome were found, as well as a what appeared to be a mutant Z chromosome variant (Z’; Fig. 2B,D).

We next employed Pacbio long-read sequencing and genome assembly on passage 31 CFS414 cells (see Supplementary Table 1 for sequencing and assembly statistics). A cont ig assembl y produced a primary pseudo-haplotype CFS414 genome of 1.05 Gb, close to the male bTaegut1.pri.v2 (1.06 Gb) and female bTaegut2.pat.W (1.12 Gb) high-quality zebra finch genome reference assemblies, generated by the Vertebrate Genomes Project (VGP)^8^. The read coverage was calculated for each chromosome by mapping raw read sequences from different assemblies onto the male bTaegut1.pri.v2 reference with the bTaegut2.pat.W W chromosome appended. For the VGP male and female raw reads, the read coverage was similarly distributed for all diploid chromosomes, peaking around the expected 88.2X for the male or 82.5X for the female sequence data (Fig. 2E; Supplementary Fig. 3A-C). As expected, the sex chromosomes were an exception, with the W chromosome absent in the male reads (Fig. 2E) and the haploid Z and W chromosomes peaking at half coverage in the female raw reads (Supplementary Fig. 3B). This analysis gave us confidence in using the genome coverage as a proxy for the mean copy number (MCN) of all chromosomes.

In contrast, the CFS414 raw read coverage distributions varied between chromosomes, indicating differences in population-level copy number (Fig. 2F). Only 15 of the 32 chromosomes had peak coverage at the MCN expected coverage level of 115X, whereas 2 were above and the remaining 15 below this level (Supplementary Fig. 3D). For example, macrochromosomes 1A, 5, 6, 8, and 9 peaked at the MCN at 115X coverage, while chromosomes 1 and 10 peaked at 140X (122% of MCN) and chromosomes 2, 3, 4 and 7 peaked at 85X (74% of MCN; Fig. 2F). While these peaks were not indicative of fully penetrant trisomies or single-copy chromosomes, they clearly showed three peak distributions in CFS414 cell populations. These findings were consistent with varying chromosome copy numbers seen in the karyotype analysis, but appeared to indicate different proportional biases than the example spreads. Overall, we believe that the coverage distributions provided a more quantitative representation for each chromosome within the CFS414 cell line.

**Figure 3:**
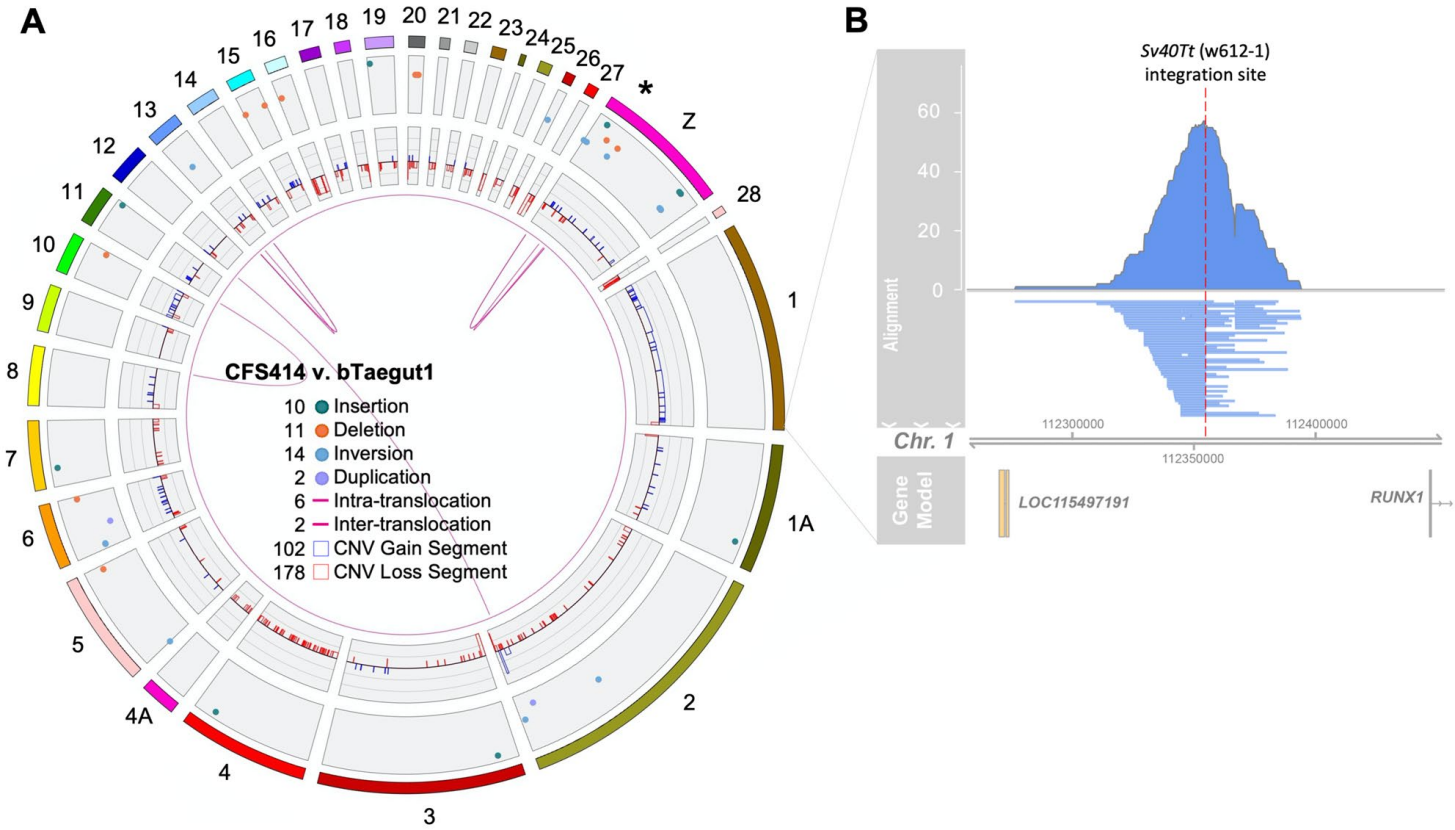
CFS414 cells are derived from a single transduction event. (**A**) Circos plot of CFS414 genome assembly compared to the bTaegut1 reference genome, showing large (≥ 150 kb) CFS414 structural variants, specified by the color legend in the center. * denotes highly variable region between the CFS414 and bTaegut1 Z chromosome. (**B**) Pileup of mapped Pacbio subreads containing the SV40Tt sequence from CFS414 cells, showing w612-1 proviral insertion in the intergenic space between *RUNX1* and *LOC115497191* on chromosome 1.

Structural variants were further determined using Bionano optical mapping data of CSF414 cells. The raw Bionano reads were assembled into a physical map and aligned to the male bTaegut1.pri.v2 reference genome^8^. From this alignment, we identified 325 large (≥ 150 kb) structural variants (indels, inversions, copy number variations, and translocations) in the CFS414 genome relative to the reference (Fig. 3A). However, most of these (280; 86%) were copy number variations, consistent with the observed aneuploidy. The gains and losses in copy numbers for each chromosome matched the raw read mapping profiles (e.g. mostly gains in chromosomes 1 and 10; mostly losses in chromosomes 2 and 4) indicating that genomic data may provide a more accurate reflection of population-wide chromosome representation in CFS414 cells than the karyotype data.

A set of particularly interesting variants in the genome was near the end of the Z chromosome, denoting several large changes possibly related to the polymorphic Z chromosome identified in the karyotypes. However, with the exception of copy number, we note that such variants are not uncommon in ‘wildtype’ genomes, as recent VGP genome assemblies have shown a higher rate of structural variation than previously believed, affecting ~ 1.3% of the genome between haplotypes or individuals^8,33^.

To determine whether these variable populations represented multiple transduction events within a polyclonal population or from a single event, subreads containing the SV40Tt proviral sequence were identified and aligned to the male reference genome to identify sites of integration. Proviral sequences were found in 115 reads, and 111 mapped to a single insertion site in chromosome 1, in the intergenic space between the *RUNX1* gene and the uncharacterized *LOC115497191* gene (Fig. 3B). The other 4 provirus-containing subreads did not align to the reference genome, likely owing to low subread sequence quality. These data suggest that, by passage 31, CFS414 cells originated from a single cell transduced with SV40Tt.

### Single-cell RNA sequencing reveals CFS414 transcriptomic landscape

To catalog the transcriptomic landscape of CFS414 cells, we used single-cell RNA sequencing (scRNAseq) for samples at passages 24 and 50 (Supplementary Fig. 4A). In all, 13,108 cells were sequenced, with a median number of ~ 5850 genes per cell identified (Supplementary Fig. 4B,C; Supplementary Tables 2 and 3 for sequence statistics). Of the 22,139 annotated genes in the genome, one or more high-confidence transcripts from 15,719 genes (71%) were detected in at least 14 cells (> 0.1%; Table 1; Supplementary Table 4). Curated gene lists were used to determine the expression of genes related to avian specialized traits or disease research. In CFS414 cells, we identified the expression of 19 genes associated with West Nile Virus infection in zebra finches^34^ and 19 gen es associated with Avian Influenza infection in chicken^35^ (Table 1; Supplementary Table 4), highlighting the possibility of exploring the role of these genes through viral infection in a robust passerine context. Of 5472 genes with specialized regulation in the zebra finch vocal learning brain nuclei compared to surrounding regions (Gedman et al., in preparation), 2967 genes were expressed in more than 10% of the cells (Table 1; Supplementary Table 4). CFS414 cells also significantly expressed 908 identified gene orthologs associated with neurological speech disorders, taken from the Human Phenotype Ontology database^37^.

**Table 1:** Expression of gene lists related to different research fields utilizing zebra finches. See Supplementary Table 4 for further expression statistics. Abbreviations: WNV, West Nile Virus; DEG, differentially expressed gene; HPO, Human Phenotype Ontology.

Genelist (# identified genes)	# Expressed ≥ 0.1% cells	# Expressed ≥ 10% cells
Zebra Finch Genes (22,139)	15,719	9915
WNV Genes (25)	19	8
Avian Influenza Genes (21)	19	15
Song Nuclei DEGs (5472)	5485	2967
*Upregulated genes* (3129)	2669	1744
*Downregulated genes* (2627)	2089	1323
HPO disordered speech genes (1184)	1090	908

**Figure 4:**
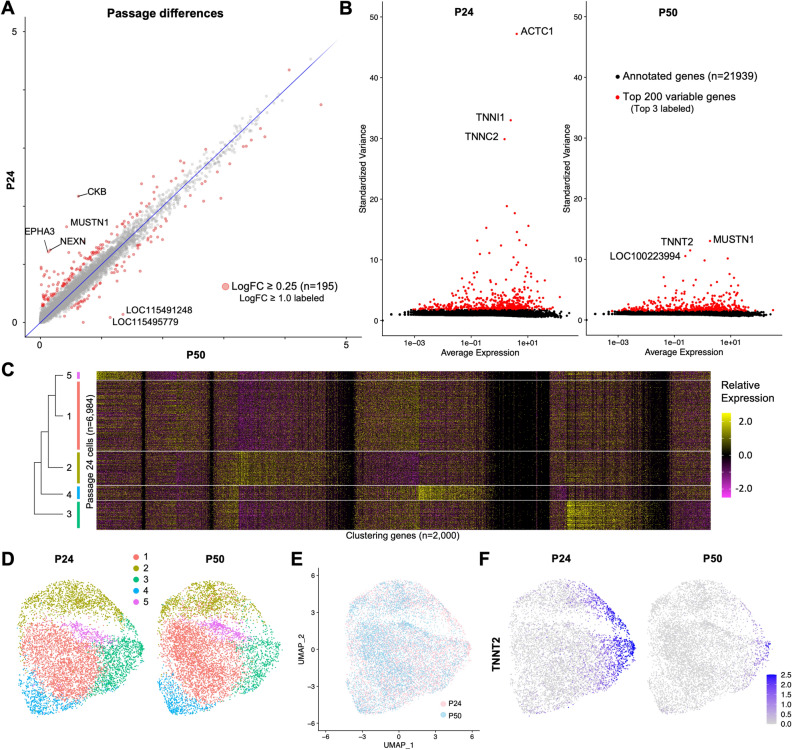
Single-cell RNA sequencing reveals relative homogeneity of CFS414 cells. (**A**) Log-transformed average gene expression (mapped reads per 10,000 cell reads) in passage 24 (P24, y-axis) versus passage 50 (P50, x-axis) cell samples. Red markers highlight a natural log fold change (logFC) ≥ 0.25; genes with logFC ≥ 1 are labeled. (**B**) Plot of all annotated genes by standardized variance (y-axis) and log-transformed average expression (x-axis) for P24 (left) and P50 (right). Note the difference in the relative decrease in variance of the top 200 genes (red) between passages. See Supplementary Fig. 4D for these plots at a y-axis scale of 0 to 5. (**C**) Heatmap of relative expression for genes used in PCA-mediated nearest-neighbor analysis, ordered by cluster relationships. On the left is a tree of cluster similarity, based on cell distances in PCA-constructed space. (**D**) UMAP plots of integrated P24 (left) and P50 (right) samples, with clusters labeled in decreasing order of cell numbers by color. (**E**) Overlay of passage 24 (pink) and passage 50 (blue) UMAP plots. (**F**) UMAP plot colored for *TNNT2* expression between passages.

We next assessed the transcriptomic stability of the cell line. We found that their respective gene expression profiles were highly concordant between passages, with only 195 genes differing by a natural log fold change (logFC) cutoff of more than 0.25 between passages 24 and 50 (Fig. 4A; Supplementary Table 4). Many of these genes (n = 126) decreased in expression between passages (Fig. 4A), and the overall cell-by-cell variability of the 200 most variable genes also declined (Fig. 4B; Supplementary Fig. 4C; P24 = 4.96 vs. P50 = 3.03; p-value = 1.54 × 10^–7^). PCA-mediated cell clustering^35,36^ of a combined passage dataset resolved 5 cell clusters (Fig. 4C, Supplementary Fig. 4E), visualized by uniform manifold approximation and projection (UMAP; Fig. 4D,E). Consistent with the genomic finding of the cell line arising from a single viral integration event in one cell, these clusters grouped into one contiguous supercluster with diffuse the boundaries between clusters. We found that many of the most variable genes decreased in one or more clusters between passages 24 and 50, such as *TNNT2* in cluster 3 (Fig. 4F). The cell number demographics of each cluster were roughly equivalent between passages 24 and 50, while over 60% of Cluster 3 cells were from passage 24 (Supplementary Fig. 4F). This difference may be due to progressive passaging modifications or minor differences in cell confluence at sequencing (Supplementary Fig. 4A,B).

**Figure 5:**
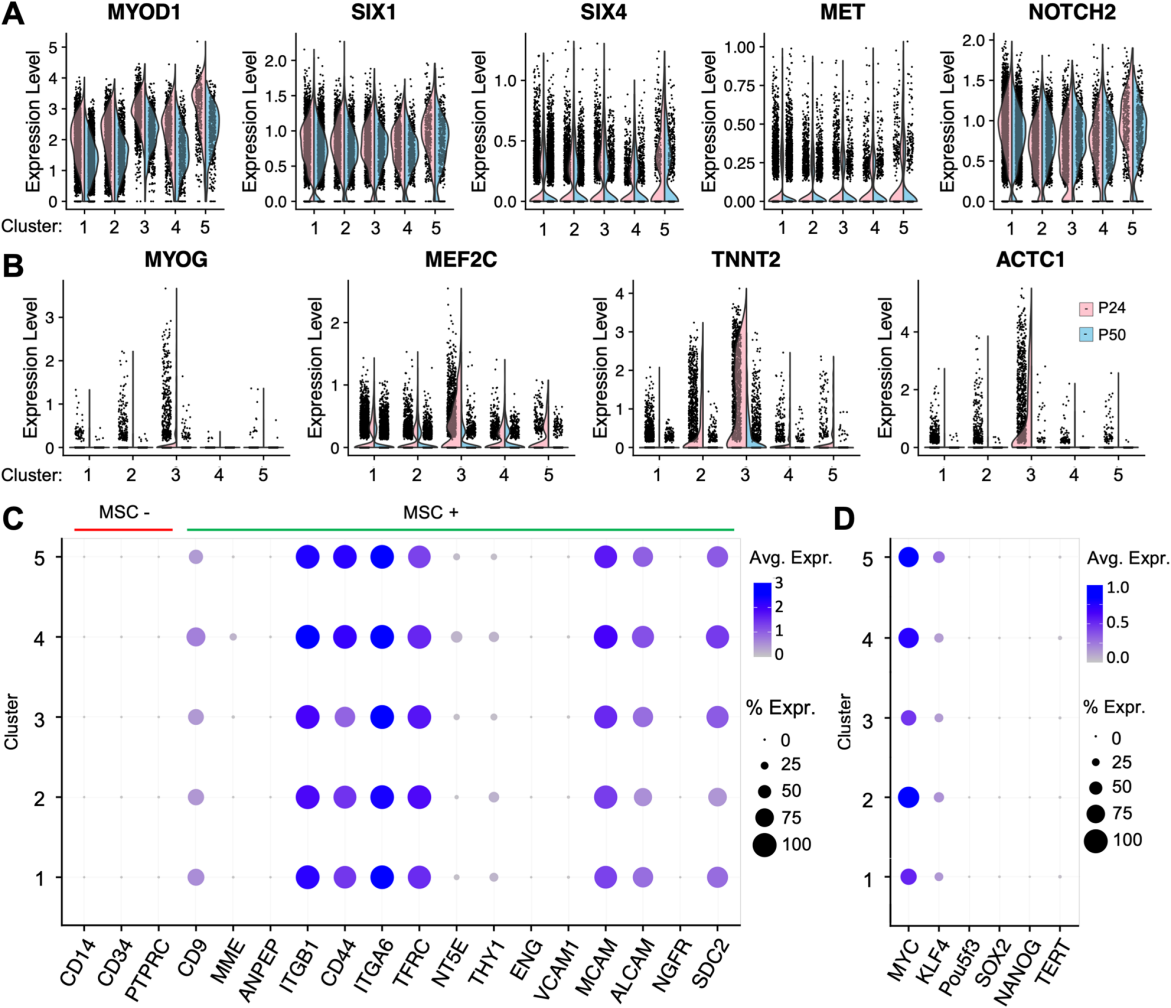
Myogenic cell identity of CFS414 cells. (**A**) Violin plots of myoblast gene markers in CFS414 cells, separated by cluster and passage. (**B**) Cluster 3-enriched myocytic gene markers. The bottom skew of the violin plots indicates cells with zero or near-zero expression. (**C**) Dot plot of negative (red line) and positive (green line) mesenchymal stem cell (MSC) markers in passage 24 CFS414 cells. (**D**) Dot plot of canonical pluripotency markers in passage 24 CFS414 cells.

A cross all clusters, gene markers for myoblasts^37^ were present (Fig. 5A), suggesting the originally transduced cell came from a myogenic lineage. The cluster tree analysis (Fig. 4C) showed that cluster 3 was the most distinct cluster, followed by clusters 4 and 2, with 1 and 5 being the least distinct. Cluster 3 differentially expressed additional myocyte markers *MYOG, MEF2C*, *TNNT2*, and *ACTC1* (Fig. 5B). For markers in clusters 1, 2, and 5, we noticed several ribosomal and cell cycle associated genes (Supplementary Table 5), suggesting these clusters represented cell state differences rather than distinct cell types. Cell cycle analy s is using markers of the DNA synthesis (S) and Growth 2/Mitotic (G2M) phases inferred a high proliferation rate across clusters, with similar proportions of cells in the S and G2M phases in clusters 1–4, with around 30% of G2M phase cells represented across both passages (Supplem entary Fig. 4G; Supplementary Table 3). Interestingly, 80% of the cells in cluster 5 were identified as being in the G2M phases, despite the exclusion of cell cycle genes to better resolve cell types during the clustering process.

To further determine the characteristics of each cluster, we performed gene ontology (GO) analyses on the upregulated gene markers of passage 24 cell clusters (Supplementary Table 6). Cluster 1, the largest, did not resolve any GO terms through its differential markers. This was largely due to the relatively small number of positive cluster markers. Cluster 2 markers suggested high metabolic activity, with terms corresponding to RNA transcription, protein synthesis, and protein localization in the membrane. Cluster 3 terms primarily corresponded to muscle function, including "muscle contraction" and "regulation of myoblast differentiation." Cluster 4 markers primarily corresponded to tissue structure and muscle fiber terms. Finally, cluster 5 was marked by “mitotic” cell division processes.

We also found that all clusters had significant expression for 10 of 15 mesenchymal stem cell markers identified in humans (Fig. 5C), a mesodermal cell type that gives rise to muscle^39^. The pluripotent stem cell markers *KLF4* and *MYC* were also highly expressed in CFS414 cells, but not *SOX2* or *OCT4* (avian ortholog *Pou5f3*; Fig. 5D). *TERT* and *NANOG* were also very low or absent in all clusters. The combined findings suggest that CFS414 cells represent an embryonic muscle stem cell population, differentiating into multiple muscle cell types, consistent with the morphologies seen in culture (Supplementary Fig. 1D). Cells from clusters 3 and 4 are potentially differentiated muscle cell types, while clusters 1, 2 and 5 likely represented transient cell states of a basal cell type.

### Disruption of zebra finch gene sequences in CFS414 cells using CRISPR-Cas9

**Figure 6:**
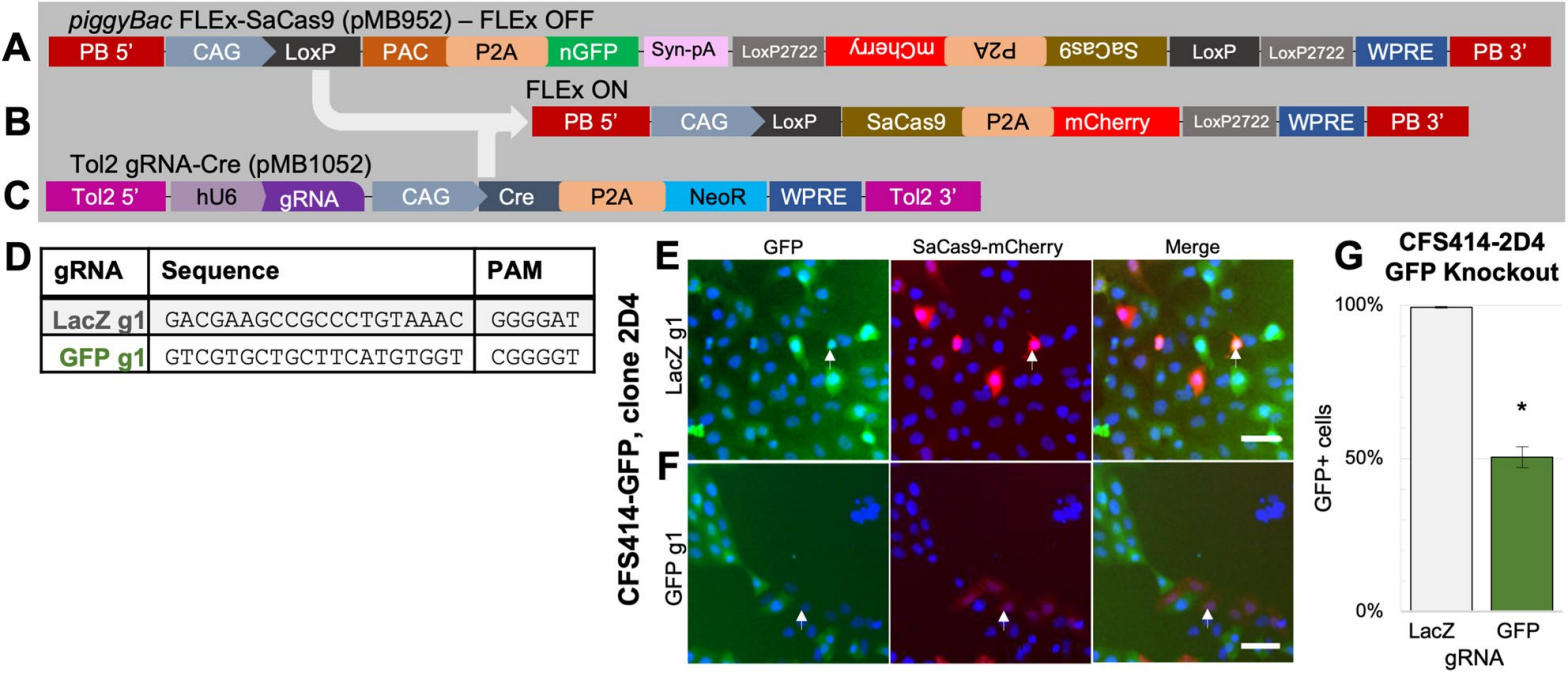
Knockout of GFP in CFS414 cells using SaCas9. (**A**) The pMB952 *piggyBac* transposon plasmid is a FLEx-cassette vector for Cre-conditional expression. Under constitutive expression, the CAG promoter expresses PAC puromycin resistance gene, with a P2A cleavage peptide sequence and nuclear GFP (nGFP). (**B**) In the presence of Cre, the PAC-nGFP sequence is excised and SaCas9-P2A-mCherry is flipped for sense expression under the CAG promoter. (**C**) The pMB1052 *Tol2* transposon plasmid is for Cre-mediated expression of SaCas9 in pMB952-integrated cells, with the delivery of a DNA-targeting gRNA. (**D**) gRNAs used to target GFP or LacZ genes with respective protospacer adjacent motif (PAM) listed. (**E–G**) The monoclonal GFP + cell line CFS414-GFP clone 2D4, transfected with *piggyBac* transposase, pMB952, and pMB1051 with (**E**) LacZ or (**F**) GFP gRNAs. (**G**) Percent of cells in LacZ and GFPg1 groups expressing GFP post-selection. * denotes significance from a two-tailed t-test, p < 0.05. Error bars denote SEM. Abbreviations: PB 5’ and 3’, *piggyBac* transposon sites; bGH pA, bovine growth hormone polyadenylation site; Syn pA, synthetic polyadenylation site.

The use of genome editing tools such as CRISPR-Cas9 has only recently been reported in songbirds^40^, in part due to the lack of robust genetic substrates for testing and optimizing these tools. To assess editing efficiency in the zebra finch genome using CFS414 cells, we generated a two-plasmid system for CRISPR-mediated gene targeting. One plasmid is a *piggyBac* transposon vector (pMB952) with a flip-excision (FLEx) cassette that expresses either nuclear GFP or *S.aureus* Cas9 (SaCas9; Fig. 6A,B). The other is a Tol2 transposon plasmid (pMB1052) containing a U6-driven gRNA cassette and Cre recombinase to activate SaCas9 expression (Fig. 6C). These vectors are selectable using puromycin or neomycin, respectively, and integrate using their corresponding transposase v ectors (Supplementary Fig. 5A,B). This plasmid system containing LacZ- or GFP-targeting gRNAs (Fig. 6D) were transfected into a CFS414 clone stably expressing GFP (clone 2D4; Supplementary Fig. 5C) and purified by antibiotic selection. The transfected gRNA vector targeting the bacterial *LacZ* gene did not affect GFP expression, whereas the gRNA targeting GFP caused significant GFP signal loss (Fig. 6E-G).

**Figure 7:**
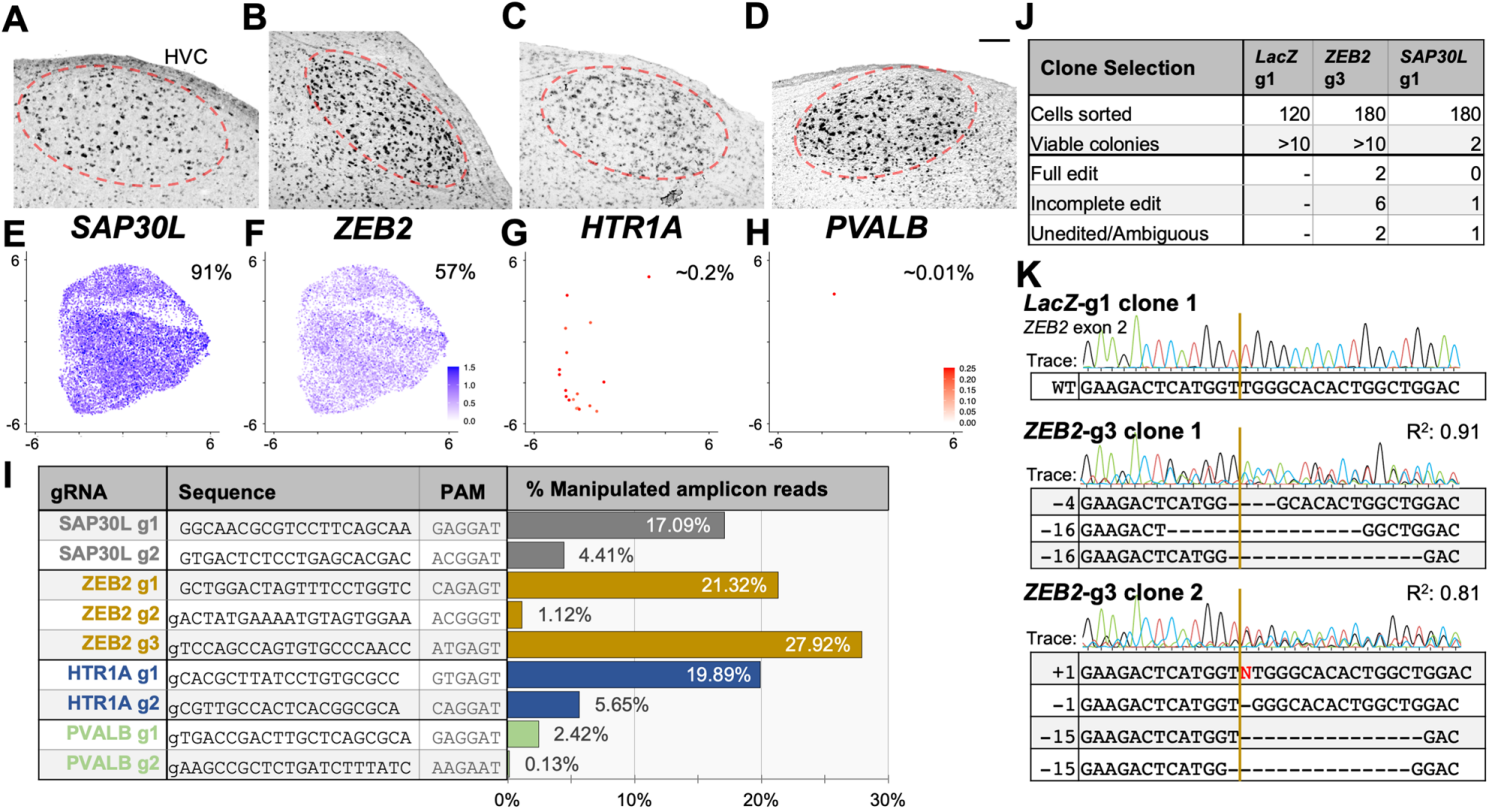
Endogenous gene sequence disruption in the CFS414 genome. (**A–D**) Chromogenic in situ hybridization images of (A) *SAP30L* (from the Zebra finch Expression Brain Atlas (ZEBrA) (RRID:SCR_012988)), (**B**) *ZEB2*, (**C**) *HTR1A*, and (**D**) *PVALB* mRNA expression in the zebra finch HVC song nucleus (red circle) under baseline conditions, provided for reference. Imaged at 10 × magnification, scale bars = 100 µm. (**E–H**) UMAP plots colored for (**E**) *SAP30L*, (**F**) *ZEB2* (**G**) *HTR1A*, and (**H**) *PVALB* expression in CFS414 cells (combined passages). The percent of CFS414 cells expressing the gene is provided below the gene name. Note the difference in scales for genes expressed at high (blue) and low (red) levels. (**I**) gRNA targets and sequences, with corresponding bar chart showing the percentage of modified reads at the target loci in CFS414-1G10 cells (Supplementary Fig. 5D–G), transfected with gRNAs targeting endogenous zebra finch genes. For specific indels identified, see Supplementary Fig. 6. (**J**) Table detailing colony viability and indel presence in gRNA-targeted clones. (**K**) Inferred sequences of fully edited clones targeted by *ZEB2*-g3, provided with *LacZ*-targeted control reference, sequence trace, and R^2^ coefficient. Brown lines denote the predicted gRNA cut site.

To assess whether Cas9 would target endogenous zebra finch genes in CFS414 cells, we generated a monoclonal cell line expressing the FLEx-Cas9 vector, denoted as CFS414-pMB952 clone 1G10 (CFS414-1G10; Supplementary Fig. 5D,E). We then transfected this cell line with Tol2 gRNA-Cre vectors targeting *SAP30L, ZEB2, HTR1A*, and *PVALB*. These genes are upregulated in the HVC song nucleus^27,41,42^ (Fig. 7A-D), which controls the timing and sequencing of song^9^. These genes differ greatly in their relative expression in CFS414 cells (Fig. 7E-H). Neomycin selection and subsequent purification by fluorescence-activated cell sorting (FACS) increased the relative population of SaCas9-mCherry-positive cells (Supplementary Fig. 5F,G). Using an established method for indel detection^43^, deep sequencing of PCR-amplified genomic loci surrounding the gRNA target demonstrated variable cutting efficiencies in 1G10 cells, with indel formations found in 0.1–27% of sequenced amplicon reads, depending on the gRNA used (Fig. 7I). Indels ranged in size from -83 to + 106 bp, though most detected indels fell within 15 bp (Supplementary Fig. 6A-M). Predicted off-target sites for 4 of the most efficient gRNAs showed very low indel formation rates, similar to untargeted *LacZ* control amplicons (Supplementary Fig. 6N). The gRNAs targeting *ZEB2* were the most effective, while those for *PVALB* were the least effective; this may be the result of intrinsic gRNA sequence or cell line properties, such as poor chromatin accessibility at the target site related to non-expression^44^. Thus, this strategy was useful in identifying gRNA candidates that induce frameshifts in the zebra finch genome.

To determine individual cell editing, we propagated clones from targeted populations. Clonal propagation of *LacZ* and *ZEB2*-g3 targeted populations resulted in several (> 10) viable colonies, while only 2 of 180 isolated *SAP30L*-g1 cells demonstrated viable colony formation (Fig. 7J). Sanger sequencing showed targeted indel formation in most clones (Fig. 7J), often with more than two indel varieties through either aneuploidy or delayed editing after clonal propagation. Two *ZEB2*-g3 clones showed full sequence manipulation through trace deconvolution (Fig. 7K), likely abrogating downstream protein expression. However, neither *SAP30L*-g1 clone contained fully edited targets. These findings suggest that SAP30L is important for CFS414 cell viability.

### Stress-responsive SAP30L localization in the zebra finch

To test the cell line’s applicability for studying zebra finch gene function, we overexpressed SAP30L. SAP30L and the SAP30 paralog interchangeably form part of the histone deacetylation (HDAC) complex^45^, engaging in binding and bending of DNA. This increases the proximity of adjacent histones and expands HDAC-mediated transcriptional repression^46^. SAP30L contains a binding domain for phosphatidylinositol monophosphates (PIPs) that overlaps with the nuclear localization signal (NLS), inducing SAP30L expulsion from the nucleus upon binding. In mammals, nuclear PIP levels are known to increase following oxidative stress^47^, making SAP30L a redox-dependent transcriptional de-repressor. SAP30L has not been studied in avian species. In the zebra finch, singing-related brain regions are characterized by high-frequency excitatory firing and NMDA receptor activation^48^. Such firing properties induce excitotoxicity and expansive oxidative stress in mice^49^. The upregulation of neuroprotective genes in song nuclei, compared to surrounding regions, has been proposed to mitigate senescence from increased activity^42,50^.

**Figure 8:**
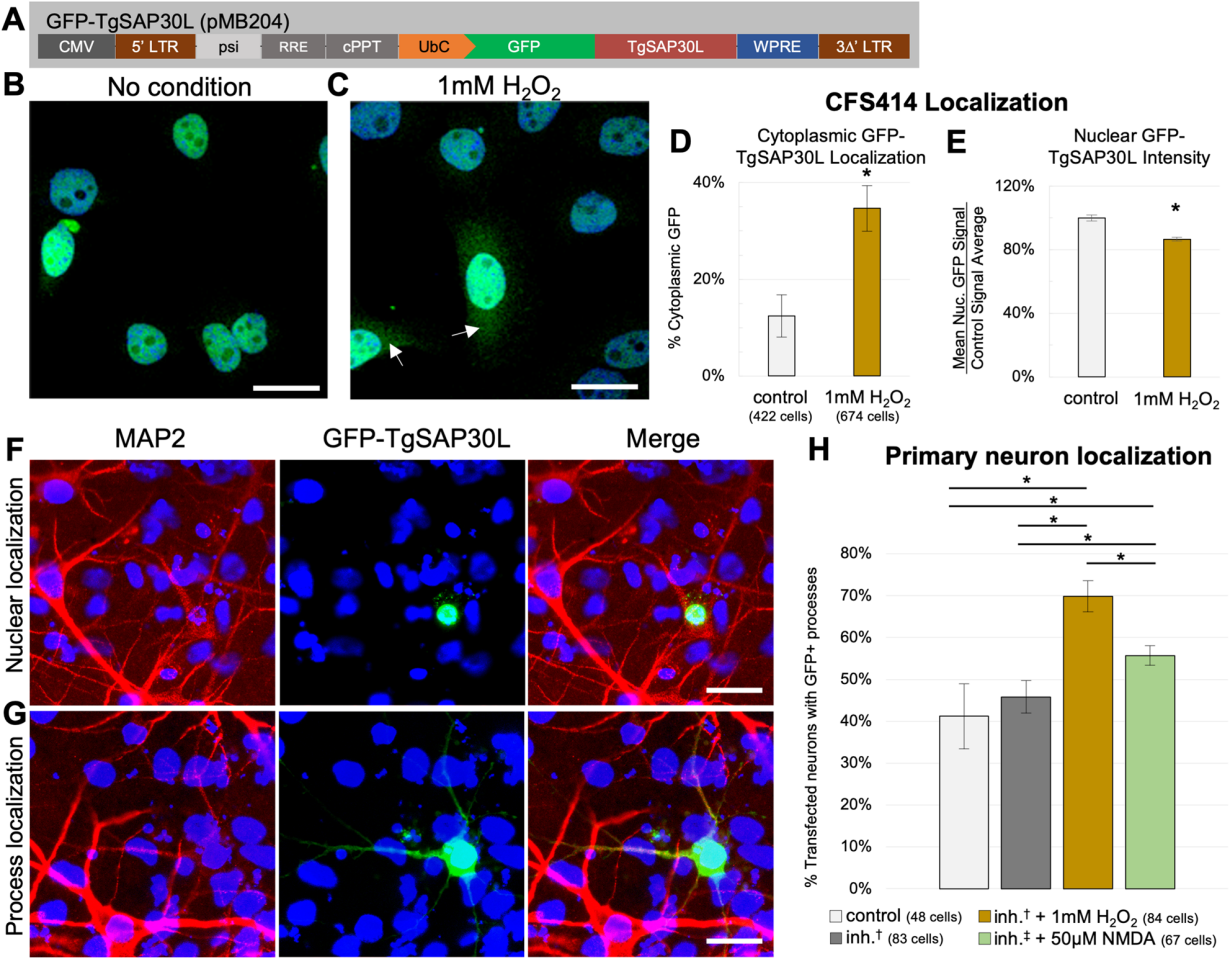
SAP30L localization in zebra finch cells is affected by oxidative stress. (**A**) Diagram of the pMB204 GFP-TgSAP30L lentiviral construct. (**B, C**) DAPI labeled CFS414-pMB204 cells under (**B**) control or (**C**) H2O2-mediated oxidative stress conditions. Arrows denote cytoplasmic localization. (**D**) Percent of CFS414-pMB204 cells showing cytoplasmic GFP signal in H_2_O_2_ and control media conditions. (**E**) Percent mean nuclear GFP signal intensity in H_2_O_2_- and control-treated CFS414-pMB204 cells, compared to the average control signal intensity. (**F, G**) MAP2 (red) and DAPI (blue) labeled primary cultured neurons transfected with pMB204 plasmid (green) showing exemplar (**F**) nuclear or (**G**) process GFP-TgSAP30L localization, both from the inhibition (inh.) conditions. (**H**) Assessment of GFP localization in control media conditions, ion channel inhibition (†, for inhibitors, see Supplementary Table 7), or challenged with either 1 mM H_2_O_2_ or 50 µM NMDA (‡, Supplementary Table 7). * denotes significance, p < 0.05 based on (**D**, **E**) two-tailed Student’s t-test or (**H**) one-factor ANOVA. Error bars denote SEM. Scale bars = 20 µm.

To assess whether SAP30L has a stress-dependent translocation response in the zebra finch, in vitro, CFS414 cells were transduced with a lentivirus containing GFP fused to the zebra finch SAP30L protein coding sequence (GFP-TgSAP30L, Fig. 8A). At baseline, these cells demonstrated strong nuclear GFP signal (Fig. 8B). When these cells were challenged with an H_2_O_2_-mediated assay of oxidative stress, they showed a threefold increase in cytoplasmic GFP signal and a concomitant decrease in nuclear GFP signal intensity compared to control cells (Fig. 8C-E). These findings indicate that the redox-dependent shuttling behavior of SAP30L is conserved between avian and mammalian cells.

To investigate the role of SAP30L in the nervous system, primary pallial neurons prepared from HH44-46 zebra finch embryos were transfected with a plasmid containing the same GFP-TgSAP30L construct. Across all conditions, transfected neurons showed differential localization on a cell-by-cell basis, with GFP-TgSAP30L localization variability seen in neuronal processes (Fig. 8F,G). Channel inhibitors did not significantly affect the proportion of cytoplasmic localization on their own, suggesting intrinsic firing was not driving cytoplasmic shuttling behavior. However, the proportion of neurons with cytoplasmic GFP did increase in both H_2_O_2_- and NMDA-challenged neurons (Fig. 8H), consistent with the H_2_O_2_-treated CFS414 results.

## Discussion

CFS414 cells overcome the obstacles of previous cell lines, fulfilling an unmet need for a robust and readily manipulable zebra finch cell line. These cells represent the first published instance of an induced cell line in any songbird species, providing insight on the cell biology of a diverse and distinct avian clade. The previous G266 and ZFTMA zebra finch cell lines^14^ have been instrumental in the study of mRNA expression^51^, antibody validation^52^, microRNA characterization^53^, and DNA methylation^54^, highlighting the importance of a zebra finch cell line. However, we and others (Carlos Lois, personal communication) have noted difficulty in maintaining these cell lines in the lab due to slow growth, low cryogenic viability, and high seeding density requirements. These previous cell lines are also not currently available through commercial or non-profit cell repositories, hindering their widespread adoption by researchers. The CFS414 and derivative cell lines are deposited with the Rockefeller University Antibody & Bioresource Core Facility for long-term storage and distribution worldwide.

The robust and versatile nature of the CFS414 cell line will enable strategies to generalize protocols and replicate findings between labs. The identification of a single SV40Tt integration point in chromosome 1 supports the origin of CFS414 cells from a single cell, likely representing the most proliferative of the transduced cells that displaced other populations across passage dilutions. Critically, density-independent proliferation is possible with this cell line, critical for monoclonal and transgenic cell line generation to improve and maintain population homogeneity.

The karyotype and sequence coverage aneuploidy seen in CSF414 cells is consistent with SV40Tt inhibiting p53^30^, a tumor suppressor involved in DNA repair^55^, potentially leading to chromosomal abnormalities. Many continuous cell lines demonstrate chromosome rearrangements, including several of the most utilized cell lines such as HeLa^29^ and DT40 cells^56^. The dynamics of SV40Tt expressed in CFS414 cells have not been directly characterized, though genome and scRNAseq analyses revealed minor variations. Nonetheless, in keeping with cell line culture guidelines^54^, we recommend maintaining frozen stocks^54^ for use only under a certain number of passages to guard against significant variation. We believe proper cell culture practices will mitigate any deleterious effects on CFS414 applications.

Originating from a male embryo, CFS414 cells are useful in the context of vocal learning research, as singing behavior in zebra finches is restricted to males. Transcriptomic analysis determined gene expression profiles suitable for the study of many genes important to vocal learning research, as well as other fields, such as avian virology and muscle function. The presence of a distinct Z chromosome variant is interesting, as several Z polymorphisms exist in zebra finch populations^15^. While it is unknown whether this structural variation originated from the embryo or from SV40Tt mutagenesis, we note that the original embryo was derived from parents with the CFW allele that present eye pigmentation deficits. Eye pigmentation deficits have been identified in juvenile zebra finches with the non-segregating "continental" chestnut-flanked white allele. While both alleles are sex-linked and non-segregating in zebra finches^58^, the genetic mechanisms have not been precisely determined. Further investigations with this cell line and animal populations may determine whether the coat-color allele and the variant Z chromosome region are related. While this cell line is not representative of the entire zebra finch genetic landscape, the demonstration of this immortalization technique may be applied to generate additional cell lines to study chromosomal polymorphisms, the female W chromosome, or the germline restricted chromosome^26^.

The CFS414 cell line expressed several gene markers for human myoblast cells, with morphological and transcriptomic evidence of active differentiation observed. The additional expression of a few pluripotent gene markers suggests that CFS414 cells exist in some potent or progenitor cell state. Previous studies have demonstrated some limited potency of myoblast cells^59,60^, and other studies have shown that SV40 T antigen expression can drive the expression of potency markers^61^. Future work can determine and optimize protocols for CFS414-derived populations to differentiate into different cell types, such as neurons^59^.

Cell lines such as CFS414 allow for the testing and optimization of tools and reagents in a *Neoaves* lineage, such as viral vectors or antibodies, before utilizing them in vivo. This was demonstrated in our use of a novel SaCas9-gRNA plasmid system, integrated into a CFS414 clone to assess targeting efficiency an d disrupt endogenous gene sequences, such as *ZEB2*. Future work may employ these fully modified *ZEB2* clones to confirm protein knockout and explore what role the ZEB2 transcription factor plays in CFS414 cells. Ultimately, preliminary validation of cutting in cell lines can build confidence before investing substantial time and resources to target these genes in vivo. The demonstration of SaCas9-mediated genome editing in zebra finches is especially promising for future studies, as SaCas9 may be packaged alongside a gRNA cassette in adeno-ass ociated viruses (AAVs) for local in vivo manipulation^10,62^.

Overexpression of zebra finch genes can identify functional roles within the context of its natural cell environment, particularly when knockout is lethal. Our SAP30L case stud y demonstrated redox-dependent shuttling into the cytoplasm, as previously documented in human cells^46^. These results were recapitulated in primary neurons, showing the novel finding of SAP30L translocation by NMDA receptor activation. SAP30L's specialized expression in several zebra finch song nuclei^41,63^ implies such cytoplasmic translocation could play a specialized role in singing behavior through activity-dependent gene de-repression. Additionally, our assays are sources of oxidative (H_2_O_2_) and excitotoxic (NMDA) cellular stress, suggesting a neuroprotective role for SAP30L in high-activity song nuclei. Future studies to conclusively determine these roles in vivo may be supported by reagent testing in CFS414 cells, further validating the cell line's utility. Other potential applications of CFS414 cells remain to be explored.

Finally, this study highlights cell line generation in non-poultry birds. While there has been evidence of insufficient immortalization from the SV40 large T antigen in another *Neoaves* species^64^, our work demonstrates the strength of SV40Tt immortalization in deriving stable zebra finch cell lines. Future work may determine the strength of this immortalization strategy in other tissues and species, both within the passerine family and across the broader avian phylogeny. Overall, CFS414 cells are a powerful resource to expand the application of emerging avian model systems.

## Methods

### Zebra Finch care and use

Animal care and experimental procedures were approved by Rockefeller University’s Animal Care and Use Committee (IACUC) and conducted in accordance with the standards set by the ARRIVE (Animal Research: Reporting of In Vivo Experiments) guidelines, American Association of Laboratory Animal Care (AALAC). Eggs were collected from coat-color validated zebra finch nests for incubation at 38 °C and 50–60% humidity. For *in* situ hybridization, we used previously collected brain tissue from adult zebra finch males (≥ 90 days old, n = 3) that were euthanized following an overnight period in a dark sound isolation chamber, as described previously^67^.

### Cell collection and culture

Hamburger-Hamilton Stage 28 (HH28)^28^ zebra finch embryos (n = 5) were removed from their eggs and euthanized by removing the head, spine, limbs and developing organs (heart, liver, mesopnephros, gonads, etc.). The rema ining embryonic skin and muscle tissue was pipetted vigorously in 0.25% Trypsin–EDTA (Gibco, Cat. #25200114) and left to incubate at 37 °C for five minutes. The tubes were centrifuged at 200×*g* for 3 min and resuspended in complete media (DMEM high-glucose, no glutamine (Cat. #11960044) with 10% (v/v) fetal bovine serum (Cat. #21640079), 1 × GlutaMAX (Cat. #35050061), 1 × Antibiotic–Antimycotic (Cat. #15240062), 1 × NEAA (Cat. #1140050), and 1 × Sodium pyruvate (Cat. #11360070), all from Gibco™). After settling, the supernatant was plated onto 10 cm dishes. Cells were incubated at 37 °C and 5% CO_2_.

To passage both primary fibroblasts and the CFS414 cells, media was removed at 100% confluence, washed with 1xHBSS (Gibco, Cat. #14170112) and trypsinized for 3–5 min. Trypsin was neutralized 3:1 with complete media, and the cells were centrifuged at 200xg for five minutes, after which the cell pellet was resuspended in complete media. Primary fibroblasts were generally passaged at a split of 1/2 and CFS414 cells at a 1/10 split, both every 4 to 6 days.

### Growth curves

For growth curve calculation, cells were seeded into a 24-well plate between 1250 cells/well (~ 1/256 dilution) and 320,000 cells/well (~ 1/2 dilution) in complete medium. For cell counting, cells were trypsinized and resusp ended in a total volume of 200–1000 µL complete media, then counted on a Countess II FL (Applied Biosystems). The log-phase doubling time, *g*, between days was calculated as:

$$\frac{{(T_{2} - T_{1} )*{\text{log}}\left( 2 \right)}}{{\log \left( {N_{2} } \right) - \log \left( {N_{1} } \right)}} = g$$

where *T*_2_ and *T*_1_ are the time at collection and the previous collection, and *N*_2_ and *N*_1_ are the averaged cell counts at collection and the previous collection time. Log phase was roughly defined as the 3 steepest consecutive slopes in the growth curve.

### Generation of lentivirus constructs and cell transduction

Plasmids used for lentiviral production may be found in Supplementary Table 8 alongside their respective sources and RRIDs. Confluent HEK293T (RRID:CVCL_0063) cells were split 1/2 into T75 flasks and transfected with molar equivalents of pMD2.G, PsPax2 and either pTK608, w612-1, or pMB204 using Lipofectamine 2000 (Invitrogen, Cat. #11668027). After three days, media was collected and passed through a 0.22 µm PVDE filter. Virus was concentrated and batches were titrated by serial dilution in HEK293T cultures, calculating approximately 1.0E7 IFU/mL for pTK608, and 1.0E9 IFU/mL for pMB204. The fluorophore-free w612-1 was assumed to be roughly equivalent to the batch-paired pTK608 titer.

Primary FEFs or CFS414 cells in 24-well plates were transduced with 10–30 µL of virus in 300 µL of complete media for 6 h, and then an additional 200 µL added to the wells 6 h later. Media was changed after 48–72 h.

### *PiggyBac* construct generation and transfection

Information on plasmids used for cloning is found in Supplementary Table 8 alongside their respective sources and RRIDs. The pMB950 and pMB1052 *piggyBac* FLEx templates were constructed by inserting synthesized gBlocks (Integrated DNA Technologies) into the PBCAG-eGFP backbone digested with *BspDI*, *SpeI*, *EcoRI*, *AgeI*, and/or *NotI* restriction enzymes (all purchased from New England Biolabs) using NEBuilder Hi-Fi Assembly (New England Biolabs, Cat. #E2621). The pMB1052 construct was subsequently cut using *BspDI* and *EcoRV* (New England Biolabs), and then cloned into a PCR-amplified pminiTol2 backbone using NEBuilder Hi-Fi Assembly. For pMB952, *S.aureus* Cas9 was amplified with homologous adaptors from pX601 by PCR for ligation into the pMB950 plasmid digested with *NheI* and *XhoI* (New England Biolabs).

CFS414 cells were transfected with Lipofectamine 3000 (Invitrogen, Cat. #L3000015) in 12-well or 6-well plates, using manufacturer’s instructions. Using vectors with antibiotic selection, cells were washed after 48–72 h after transfection with complete media containing 0.5 µg/mL Puromycin (Gibco, Cat. #A1113802) or 1.5 mg/mL Geneticin (Gibco, Cat. #10131035), with antibiotic media replaced every three days until the desired selection level was reached. *PiggyBac* transposon vectors were co-transfected with the pCyL43 *piggyBac* transposase vector^68^. Tol2 transposo n vectors were co-transfected with pCMV-Tol2.

### Genomic loci amplification

Genomic DNA was extracted from cells using a routine genomic DNA extraction protocol (cell lysis, RNA digestion, protein precipitation, and subsequent alcohol washes) prior to DNA resuspension in Ultrapure distilled water (Invitrogen, Cat #10977015). Genomic loci were then amplified using primers targeting genes of interest (see Supplementary Table 9 for a list of primers) using Q5 Hot Start High-fidelity 2X Master Mix (NEB, Cat. #M0494). One exception was the *CHD* sextyping amplification protocol, which followed a Taq polymerase protocol outlined previously ^69^.

### Karyotyping

CFS414 cells were seeded at 1/4 dilution in T-25 flasks and shipped overnight to KaryoLogic, Inc. (Durham , NC), where Giemsa-banded karyotyping was performed as previously described^13^.

### Pacbio gDNA sequencing, assembly, and structural variant analysis

Roughly 4 million CFS414 cells were pelleted and stored at -80 °C. Ultra-high molecular weight (uHMW, ~ 50–300 kb) DNA was extracted using the Nanobind CBB Big DNA Kit (Cells, Bacteria, Blood; Circulomics, Cat. #NB-900-001-01). uHMW DNA quality was assessed by a Pulsed Field Gel assay and quantified with a Qubit 2 Fluorometer, and 5 µg of uHMW DNA was sheared using a 26G blunt end needle (Pacbio protocol PN 101-181-000, Version 05). A large-insert (23 kb average) Pacbio library was prepared using the Express Template Prep Kit v1.0 (Pacific Biosciences, Cat. #101-357-000) following the manufacturer’s instructions. Library size selection using the Sage Science BluePippin Size-Selection System was made for inserts larger than 20 kb. This library was sequenced with a Pacific Biosciences 8 M (Cat. #101-820-200) SMRT cell on the Sequel II instrument with the Sequencing Kit 2.0 (Cat. #101-820-200) and Binding Kit 2.0 (Cat #101-842-900), recording a 15-h movie of sequence reads, producing Continuous Long Reads (CLR). Raw Pacbio subreads were mapped to the zebra finch reference genome bTaegut1.v2 (with W chromosome from bTaegut2.pat added) using minimap2^70^. Base coverage was calculated using SAM tools 1.9^71^ and BEDtools genomecov v2.27.1^72^.

Pacbio subreads were filtered using BLAST^73^. Subreads that have a significant overlap with the w612-1 proviral sequence were selected and mapped to the reference genome using minimap2 and bam files were sorted and indexed using SAMtools. Alignment was visualized in IGV^74^ and plotted using R custom scripts.

A total of 13,928,268,101 bp of sequence was generated with a subread N50 of 33,751 bp. The genome was assembled using Falcon-unzip 2.2.4^75^, with primary contigs filtering for artifacts (haplotypic duplications) using Purge-dups^76^. The final primary assembly length was 1,047,588,919 bp, composed of 356 contigs (N50 contig = 12,971,239 bp).

### Bionano structural variant analysis

uHMW DNA was labeled for Bionano Genomics optical mapping using the Bionano Prep Direct Label and Stain (DLS) Protocol (Cat. #30206E). To image, labeled DNA was run on a Saphyr instrument chip flowcell. 420.67 Gb (read length ≥ 150 kb) was generated with read length N50 = 343.5 kb and average label density of 13.92 labels per 100 kb. A consensus genome map was constructed and aligned to the zebra finch reference genome using Solve 3.6.1_11162020. The Variant Annotation Pipeline was used to identify signatures of structural variants.

### Single-cell RNA sequencing

CFS414 cells at passage 24 and passage 50 were trypsinized and counted on a Countess II FL (Life Technologies), and a single cell suspension was diluted to around 1000 cells/µL in 1xPBS. Approximately 5000 cells were run on a Chromium Chip (10 × Genomics). cDNA synthesis and library preparation were performed using the Chromium Single Cell 3’ Reagent Kits (version 3, 10 × Genomics) according to manufacturer’s instructions. Sequencing was performed by Novogene Co., Ltd. on an Illumina HiSeq 4000.

Raw sequencing data was aligned to a VGP male zebra finch genome assembly (bTaegut1.pri.v2), with the addition of the W chromosome from bTaegut2.pat.W, using the CellRanger analysis pipeline (10 × Genomics, version 3) to pre-process and filter the dataset for high-confidence read mapping. The 3’ and 5’ UTRs of annotated genes were extended using previously collected bulk RNAseq data to enhance read alignment. Analysis of the sequence data was performed using the Seurat tools workflow (version 3.2.0)^65^ in RStudio (version 4.0.2). Briefly, the dataset was further trimmed, normalized, and scored for cell cycling to regress zebra finch genes and Refseq IDs associated with G1, G2M and S phases, as well as mitochondrial genes. These datasets were then scaled by SC-Transform^66^ using Seurat commands. The two datasets were integrated using anchor-based nearest neighbor functions using default settings, and 13 PCAs using the standard cutoff (2000) of the most variable genes identified were used to calculate the clusters. A detailed breakdown of analysis may be found on Github (https://github.com/Neurogenetics-Jarvis/CFS414_scRNAseq). For specific gene visualization and DEG identification, expression was defined as a log1p-transformation of the number of transcripts per 10,000 barcode reads. Average expression for each gene is listed by passage in Supplementary Table 4. GO terms for clusters were found using the GOrilla webtool^77^ with a single, ranked list of gene markers (adjusted p-val ≤ 0.05; LogFC ≥ 0.1).

### Fluorescence-activated cell sorting and clonal propagation

Cells were trypsinized and resuspended in cell sorting buffer (1 × DPBS (Ca/Mg++, pH 7.0–7.4), 10 mM HEPES (Gibco, Cat. #15630080), pH 7.0, 0.1% Bovine Serum Albumin, (Invitrogen, Cat. #AM2616), 5 mM EDTA, and 2–8 ng/mL DAPI (4′,6-diamidino-2-phenylindole, used as a vital stain)), passed through a 40 µm filter and diluted to 1.0–3.0E6 cells/mL. Cells were taken to the Rockefeller Flow Cytometry Resource Center and loaded into a FACS Aria II (BD Biosciences). Live (DAPI-free) cells were sorted by desired fluorescent intensity. Samples were either sorted in bulk (purified) or single (clonal) cells, index-sorted into a 96-well plate containing 100µL conditioned, filtered complete media from CFS414 cells. Purified cells were washed twice with complete media with 5 × antibiotic–antimycotic and plated. For clonal samples, plates were incubated at 37 °C, 5% CO_2_. After 11 days colony formation was assessed and suitable clones were harvested for expansion.

### Single-label in situ hybridization

Digoxigenin- (DIG) conjugated probes for *ZEB2* (Genbank: DV946921) and *PVALB* (Genbank: DQ215755) were generated and applied onto male zebra finch brain sections from previous studies, as detailed previously^67^. For the *HTR1A* DIG-labeled probe, the sequence was amplified from zebra finch cDNA using pri mers containing the T7 and T3 promoters (Supplementary Table 9), and the probe was generated and used in the same way as the others.

### CRISPR-gRNA cutting assays

The LacZ and GFPg1 gRNAs were selected for minimal zebra finch genome sites for potentia l off-target cutting using GT-Scan^78^. Endogenous genes were targeted using gRNAs designed by GT-Scan or CRISPOR^79^. For gRNA sequences, see Fig. 7I and Supplementary Fig. 6. Oligonucleotides (Integrated DNA Technologies) of the gRNA sequence were then annealed and ligated into the pMB1052 vector, using a protocol for the AAV construct, pX601 (https://www.addgene.org/crispr/zhang/). Cells were lipofected with pMB952, pMB1052, and pCyL43 and purified with Geneticin 48 h later (Gibco, Cat. #10131027). Prior to imaging, cells were washed with complete media containing 100 ng/mL Hoechst 33,342 (Invitrogen, Cat. #H3570).

CFS414 clone 1G10 cells with the transposon-integrated pMB952 construct were transfected with pMB1052 constructs containing gRNAs targeting endogenous zebra finch genes (for sequences, see Fig. 6J). Cells were selected with 1.5 mg/mL Geneticin (Gibco, Cat. #10131035) and further purified by FACS. Genomic DNA was extracted, and the genomic loci surrounding the gRNA or predicted off-target sites was amplified by PCR (see Supplementary Table 9 for primers). Illumina N7xx and N501 adaptors were added by fu rther PCR amplification^80^. Primers were removed using AMPure XP beads (Beckman Coulter, Cat. #A63880), and the amplicon quality was assessed on an Agilent 2100 BioAnalyzer before samples were pooled into a 10 µM DNA library. DNA libraries were to run on a MiSeq benchtop sequencer (Illumina) according to manufacturer’s instructions. Indel formation was assessed and visualized using CRISPResso2^81^.

FACS-purified cells were re-sorted for clonal propagation in high-efficiency gRNA-targeted samples with endogenous target gene expression. Colonies were considered viable if they were large enough for clonal propagation after 21 days. Sanger sequencing of target amplicons was performed and sequence trace files were analyzed using the Synthego ICE Analysis Tool (v3.0)^82^. "Complete knockouts" were considered to be clones with no inferred wildtype sequences; "incomplete knockouts" were clones with 25%-99% edited sequence representation; "unedited/ambiguous" were clones with less than 25% of edited contributions identif ied.

### GFP-SAP30L cytoplasmic shuttling assay

CFS414 cells were transduced with pMB204, showing variable expression level likely based on proviral integration differences. These cells were then plated onto coverslips and allowed to grow for 24 h. Conditioned media was removed and the cells were challenged with 0.0 mM or 1.0 mM H_2_O_2_ in fresh culture media for 15 min, after which the conditioned media was replaced. After a 4 h recovery period, cells were washed with 1xPBS and fixed for 15 min in in 4% PFA in 1xPBS. DAPI nuclear counterstain was conducted and the slides coverslipped with ProLong Diamond Antifade mounting medium.

### Zebra finch primary neuron culture

For the culture of zebra finch neurons, an adapted protocol of m ouse cortical neuron culture was used^83^. Briefly, Hamburger-Hamilton stage 45 embryos or day 0 hatchlings were euthanized, followed by brain dissection and the removal of meninges. Hemispheres were approximately resected to remove the midbrain and basal ganglia; the remaining pallial regions (i.e., hyperpallium, mesopallium, nidopallium, arcopallium) were placed in 200 arbitrary units/mL papain (Worthington, Cat. #3126) for 5–7 min and neutralized with trypsin neutralization solution (Sigma, Cat. #T9253). Cells were plated onto Laminin-treated coverslips (Neuvitro, Cat. #GG-12-Laminin) at a density of 50,000 cells/cm^2^ in cortical plating media (Basal Medium Eagle (Sigma B1522), 0.6% Glucose, 10% (v/v) fetal bovine serum, 1 × GlutaMAX, 1 × Antibiotic–antimycotic). One day after plating, media was removed and replaced with serum-free neurobasal media (Gibco, Cat. #21103049) with 1 × B27 supplement (Gibco, Cat. #17504044), 1 × GlutaMAX, and 1 × Antibiotic–antimycotic.

Neurons were transfected at 6 days in vitro (DIV6) with pMB204 plasmid using a mixture of Lipofectamine 3000 and Combi-Mag reagent (OZ Biosciences, Cat. #KC30200). Variable GFP expression levels were noted across neurons, likely owing to plasmid copy number or neuron cell type but assumed to be similarly variable across wells at baseline. At DIV8, cells were left untreated or treated with channel inhibitors cyanquixaline (CNQX, Sigma, Cat. #C127), Bicuculline (Sigma, Cat. #14340), tetrodotoxin (TTX, Abcam, Cat. #ab120054), and DL-2-amino-5-phosphonopentanoate (APV, Sigma, Cat. #A5282) (Supplementary Table 7). After 16 h, neurons were challenged with fresh media with or without inhibitors for 3 h, 1 mM H_2_O_2_ for 15 min, or 50 µM NMDA for 1 h, then allowed to recover for 2 or 4 h in their corresponding conditioned media. The fresh media timepoint was selected based on a 3-h KCl depolarization assay that showed no effect.

### Immunocytochemistry

Neurons on coverslips were fixed with 4% ice-cold PFA, washed with 1xPBS, and permeabilized with 0.5% Triton-X in 1xPBS. Prepared slides were then blocked in 10% Blocking One (Nacalai, Cat. #0395395), 0.5% Triton-X, and 1xPBS for 30 min, then incubated with 1:1000 primary antibody (Chicken anti-GFP (Sigma, Cat. #AB16901, RRID:AB_11212200) and Mouse anti-MAP2 (Sigma, Cat. #MAB3418, RRID:AB_94856) overnight at 4 °C. Sections were then washed 3 times with 1xPBS with 1% Tween-20 (PBS-T) for 15 min and then transferred to host-specific 1:200 fluorophore-conjugated secondary antibody (Goat anti-Chicken 488 (ThermoFisher, Cat. #A11039, RRID:AB_142924) and Goat anti-Mouse (ThermoFisher, Cat. #A21125, RRID:AB _141593)) for one hour at RT. Cells were then washed again with 1xPBS-T and DAPI nuclear counterstain was applied for imaging.

## Quantification and statistics

All genomic, transcriptomic, and amplicon dataset quantification and statistical analysis was performed as described above.

For the CFS414-GFP clone 2D4 gRNA targeting experiments, cell nuclei were counted in FIJI (n = 4 images per condition), then co-localized with GFP signal to determine percentage of total cells. Analysis was unblinded, as signal loss was visibly apparent without quantification. Significance was determined by a Student’s two-tailed, equal variance t-test.

For the CFS414 cytoplasmic shuttling assay, samples were imaged on a Zeiss LSM 780 laser scanning confocal microscope. To determine cell localization, non-transduced cells (< 1% total population) were identified and used to normalize for background signal in the image (n = 4 images per condition). Images were blinded for analysis, and DAPI signal was subtracted from the GFP signal in transfected cells, and cytoplasmic GFP signal in the remaining cells was counted using Adobe Photoshop (v22.0.1, RRID: SCR_014199). The mean grey value in the GFP channel was also calculated for each nucleus using the Measurement tool. Significance was determined using Student’s two-tailed, equal variance t-tests.

To determine cell localization of GFP in primary neurons, several images were collected per condition using an Olympus BX61 upright microscope. Images (n = 6–8 images per condition) were blinded for analysis, and GFP signal co-localized with blue DAPI signal and surrounded by MAP2 cytoskeletal marker was identified as neuronal expression. An average of 9.7 transfected neurons were identified in each image. GFP signal that extended beyond the DAPI-stained nucleus and into the MAP2-stained processes was considered cytoplasmic localization. A percentage of cytoplasmic localization was calculated per image and averaged for each condition. Significance was calculated with a one-factor ANOVA with a post-hoc Tukey–Kramer test.

## Supplementary Information

Supplementary Information 1.

Supplementary Information 2.

## Data Availability

Genome sequence data is available through NCBI BioProject PRJNA789356. Single-cell RNA sequencing data is available through NCBI GEO (Accession: GSE195934), and code for Seurat processing and figure generation may be found on Github (http://github.com/Neurogenetics-Jarvis). Further requests for datasets generated should be directed toward the corresponding authors (mbiegler@rockefeller.edu, ejarvis@rockefeller.edu).

## Materials availability

The CFS414 cell line (RRID:CVCL_A7AT) and select clones are available through the Bi-Institutional Antibody and Bioresource Core Facility of Memorial Sloan Kettering Cancer Center and The Rockefeller University (RRID:CR_017691; https://www.rockefeller.edu/monoclonal/cell_line_distribution/), and other clones are available upon request. Plasmids generated are available through Addgene or otherwise by request to the corresponding authors.
