# Supplementary material for: Induction of an immortalized songbird cell line allows for gene characterization and knockout by CRISPR-Cas9
Source: Sci Rep. 2022 Mar 14;12:4369. doi: 10.1038/s41598-022-07434-7 (PMC8921232; doi:10.1038/s41598-022-07434-7)
Supplement: Supplementary file 1 — Supplementary Information 1. [file 41598_2022_7434_MOESM1_ESM.pdf]

Induction of an immortalized songbird cell line allows for gene characterization and knockout by CRISPR-Cas9

Matthew T. Biegler*[1,2], Olivier Fedrigo[3], Paul Collier[4], Jacquelyn Mountcastle[3], Bettina Haase[3], Hagen U. Tilgner[4], Erich D. Jarvis*[1,2]

[1]Laboratory of Neurogenetics of Language, The Rockefeller University, New York, NY, 10065, USA
[2]Howard Hughes Medical Institute, Chevy Chase, MD, USA
[3]Vertebrate Genome Laboratory, The Rockefeller University, New York, NY, 10065, USA
[4]Center for Neurogenetics, Graduate School of Medical Sciences, Weil Cornell Medical Center, New York, NY, 10065, USA

SUPPLEMENTARY FIGURES

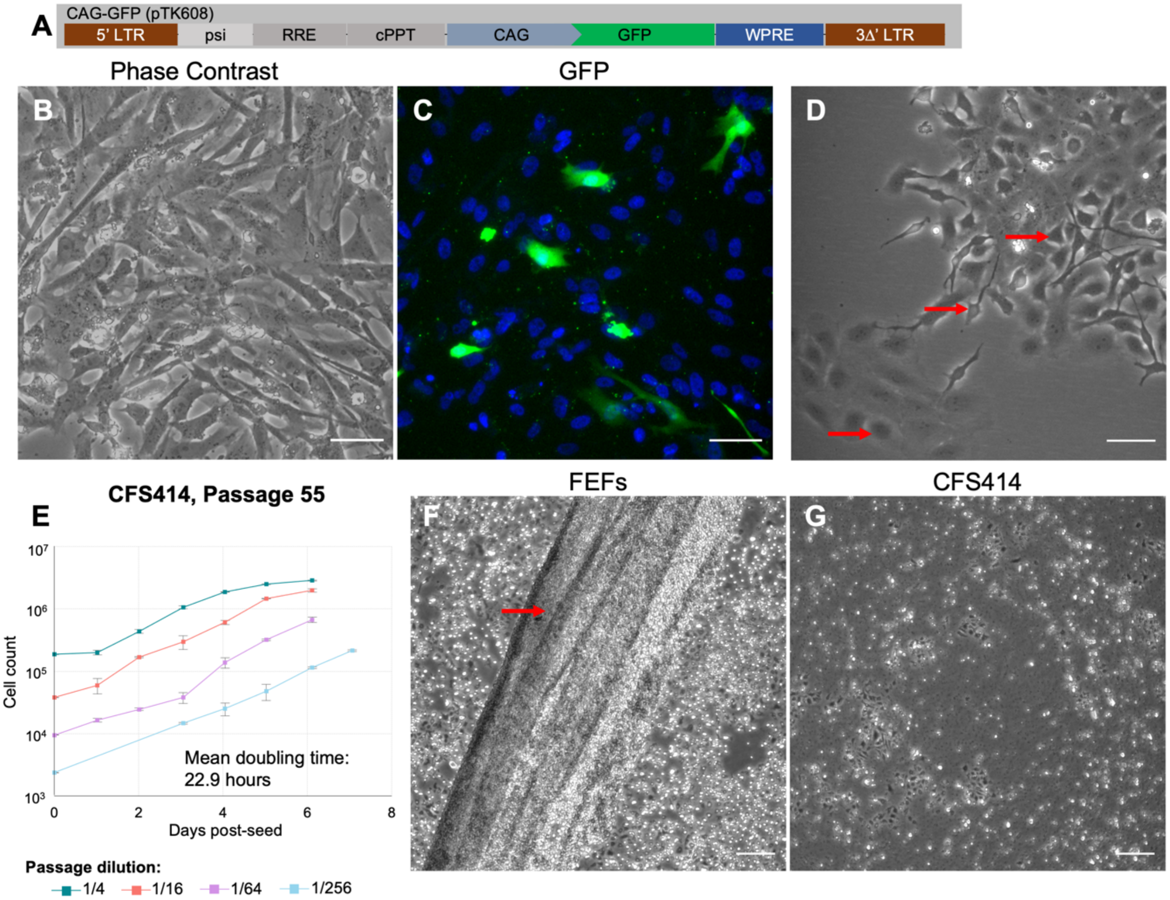

Supplementary Fig. 1. Zebra finch FEF and CFS414 characterization.
(A) Diagram of CAG-GFP control lentiviral construct. (B-C) Passage 2 Finch embryonic fibroblasts (FEFs) 72 hours following transduction with CAG-GFP (pTK608) control lentivirus imaged by (B) phase-contrast and (C) fluorescence to show sparse GFP (green) transduction with Hoechst 33342 nuclear counterstain (blue). (D) Brightfield image of CFS414 cells plated at low seeding density, exemplifying the morphological diversity present. Note the slender cells showing processes as well as the flatter, more square cells (red arrows). (E) Growth curve of passage 55 CFS414 cells at variable seeding densities, ranging from a dilution of 1/4 (160,000 cells/well) to 1/256 (1,250 cells/well). Error bars, SEM. (F) Exemplary image of cell sheet formation (red arrow) by overconfluent FEF control cells that have been trypsinized. (G) Similarly overconfluent, trypsinized CFS414 cells for comparison. Scale bars (B-D) = 50μm; (E-F) = 200μm.

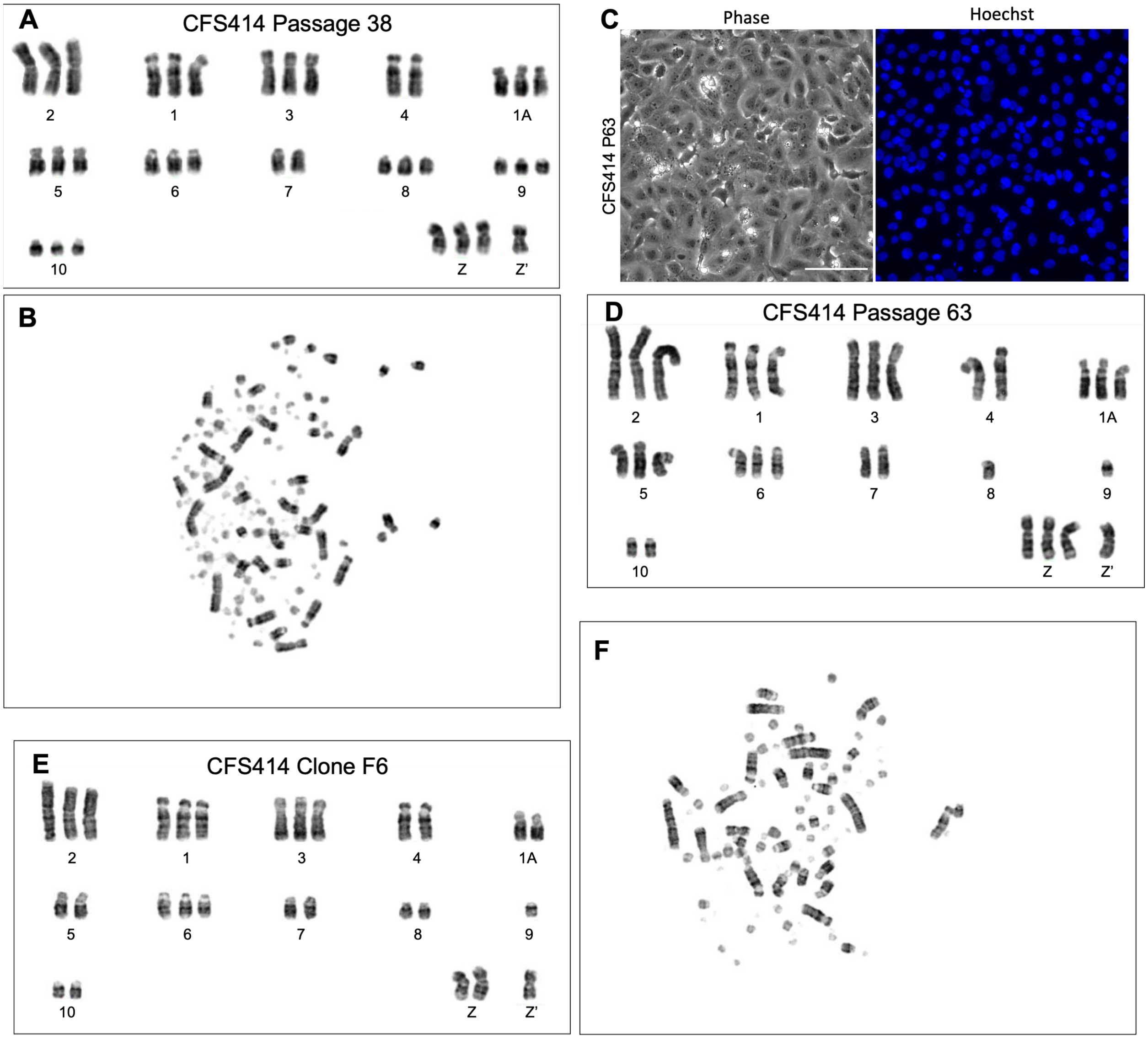

Supplementary Fig. 2. Karyotypes of CFS414 passage and clonal cell populations.
(A) Second exemplar karyotype of passage 38 CFS414 cells. (B) Corresponding metaphase spread for (A). Note the presence of unsorted microchromosomes. (C) Phase contrast and Hoechst 33342 stained images of passage 63 CFS414 cells. Scale bar = 50μm. (D) Example karyotype of passage 63 CFS414 cells. (E) Second exemplar karyotype of CFS414 clone F6 cells with (F) Corresponding metaphase spread for (E). Note presence of mutant Z chromosome (Z') in all karyotypes.

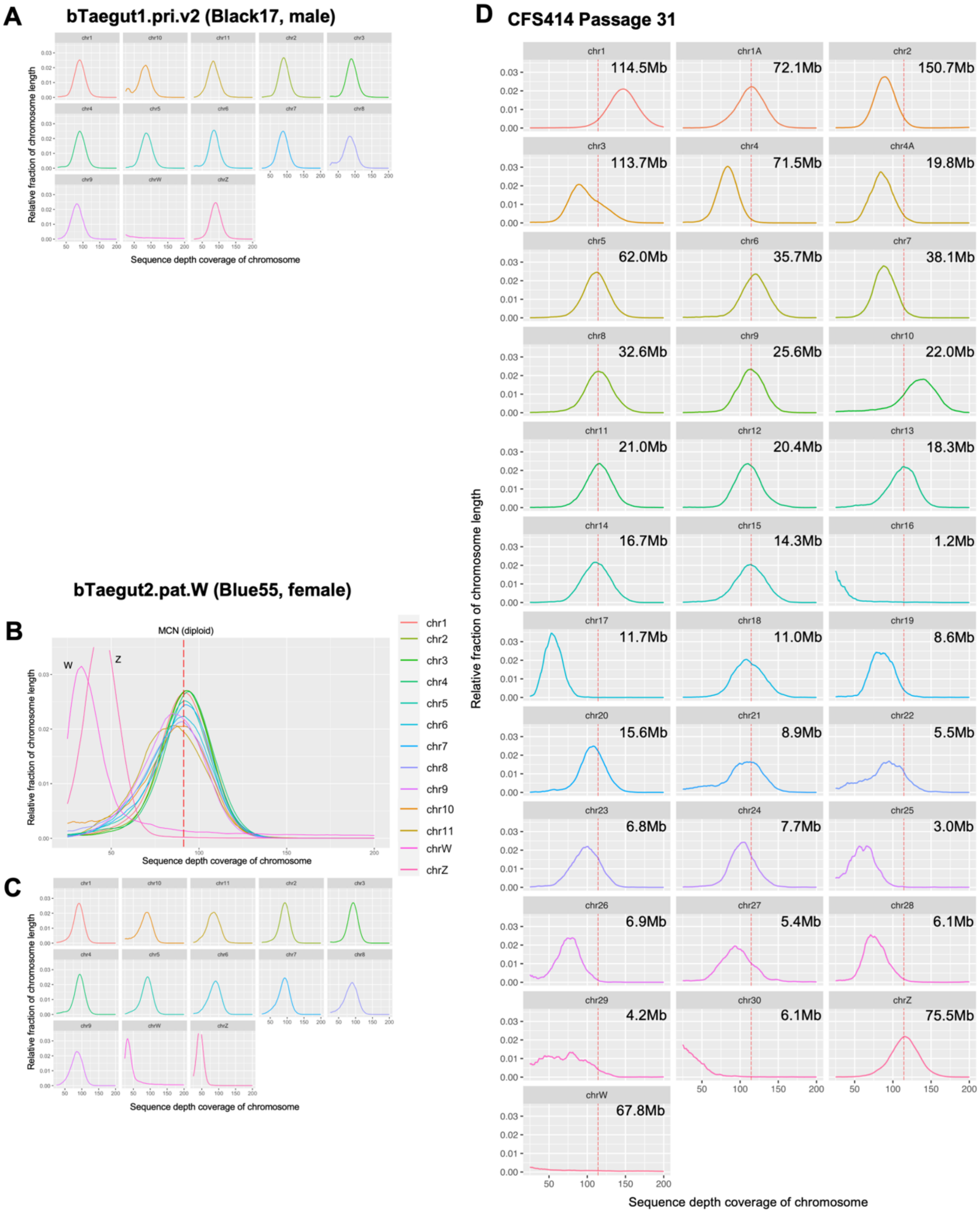

Supplementary Fig. 3. Coverage variability within the CFS414 genome assembly.

(A) Individual charts of fraction of chromosome coverage of all chromosomes, listed with the assembled chromosome size from bTaegut1.pri.v2 and red dotted lines delineating the MCN. Aberrant microchrosomes may be due to difficulties mapping repetitive sequences. (B) bTaegut1 macrochromosome coverage chart, separated. (C) Coverage plot of the bTaegut2.pat.W female reference genome (coverage 82.5x), highlighting the haploid sex chromosomes. (D) Separation of coverage peaks in part C.

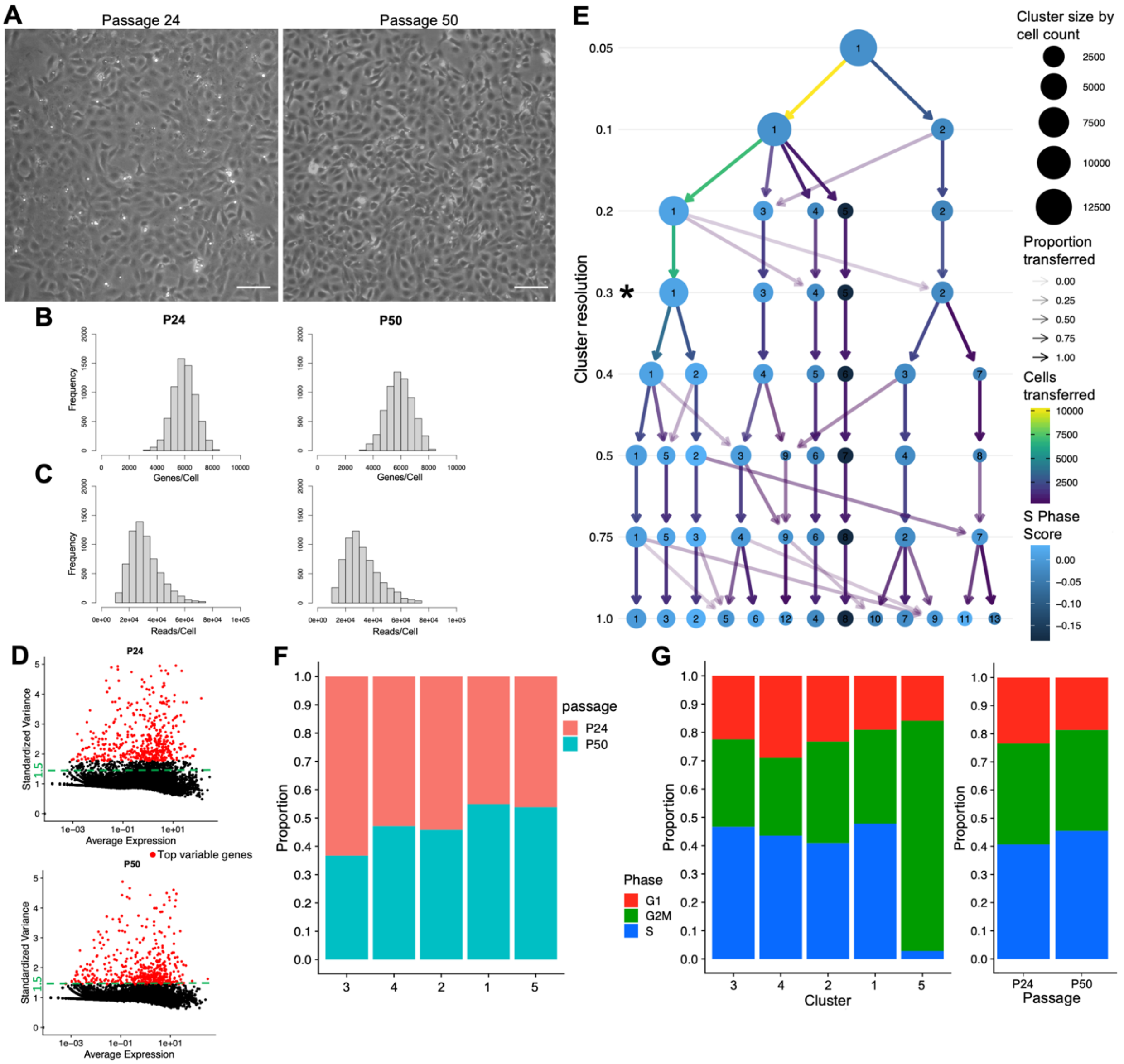

Supplementary Fig. 4. Single-cell RNA sequencing data

(A) Phase contrast images of CFS414 cell samples prior to trypsinization for scRNAseq. Scale bars = 100μm. (B-C) Histograms of (B) Feature counts (Genes/cell) and (C) Raw RNA counts (Reads/cell) for passage 24 (P24, left) and passage 50 (P50, right) CFS414 cell samples. (D) Plot of all annotated genes by standardized variance (y-axis) and average expression (x-axis) for P24 (left) and P50 (right). Subset of Fig. 3B (y-axis from 0 to 5) to emphasize difference in top genes by standardized variance (red) between passages. (E) Cluster tree of scRNAseq object used to select the clustering resolution (i.e. cluster formation sensitivity), showing cluster and cell designation changes over resolution values. Cluster nodes are colored by S Phase cell cycle score and sized by total cell population. Arrow color and transparency the respective number and proportion of cells transferred between clusters across resolutions. * highlights chosen resolution (0.3) for downstream analysis based on cluster stability and cell cycle homogeneity. (F) Proportional bar chart of cluster makeup by passage (H) Proportional bar chart of cell cycle phase (determined through scRNAseq processing), split by cluster (left) and passage (right). Abbreviations: G1, Growth 1 phase; G2M, Growth 2 and Mitotic phases; S, synthesis phase.

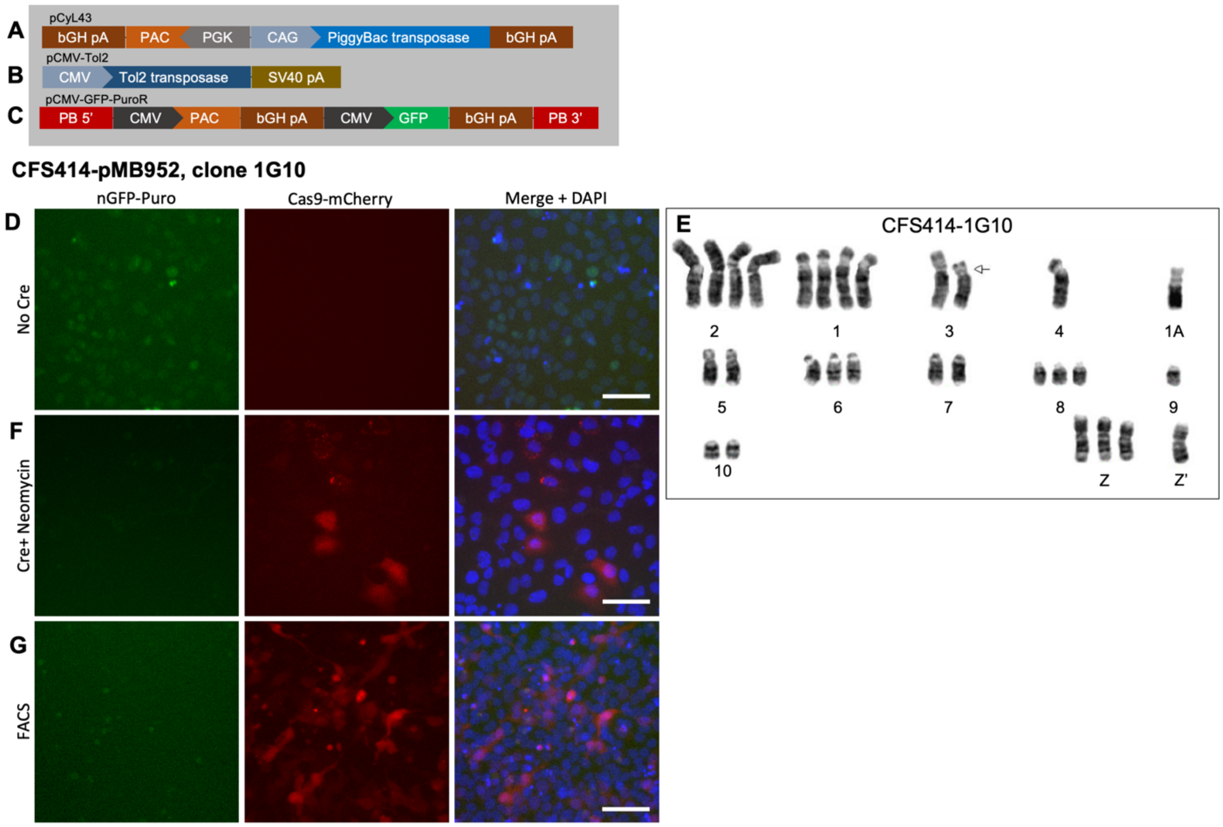

Supplementary Fig. 5. Function of pMB952 and pMB1052 vectors.
(A) *PiggyBac* transposase vector pCyL43. (B) *PiggyBac* transposon vector expressing GFP and the Puromycin resistance gene, Puromycin N-acetyltransferase (PAC). (C) *Tol2* transposase vector pCMV-Tol2 for transfection with pMB1052, Fig. 6B. (D) CFS414 clone 1G10 cells integrated with the pMB952 plasmid. Nuclear green (nGFP) signal from the unrecombined pMB952 plasmid, overlaid with Hoescht33342 nuclear stain (blue). (E) Exemplary karyotype of CFS414 clone 1G10 cells at passage 9. Arrow denotes partial deletion in chromosome 3. (F) Cre expression through pMB1052 transfection into some cells conditionally excised nGFP signal and expressed SaCas9-P2A-mCherry. Geneticin treated to reduce untransfected cells. (G) FACS-mediated population of 1G10 cells, showing high mCherry populations. Scale bars = 50μm.

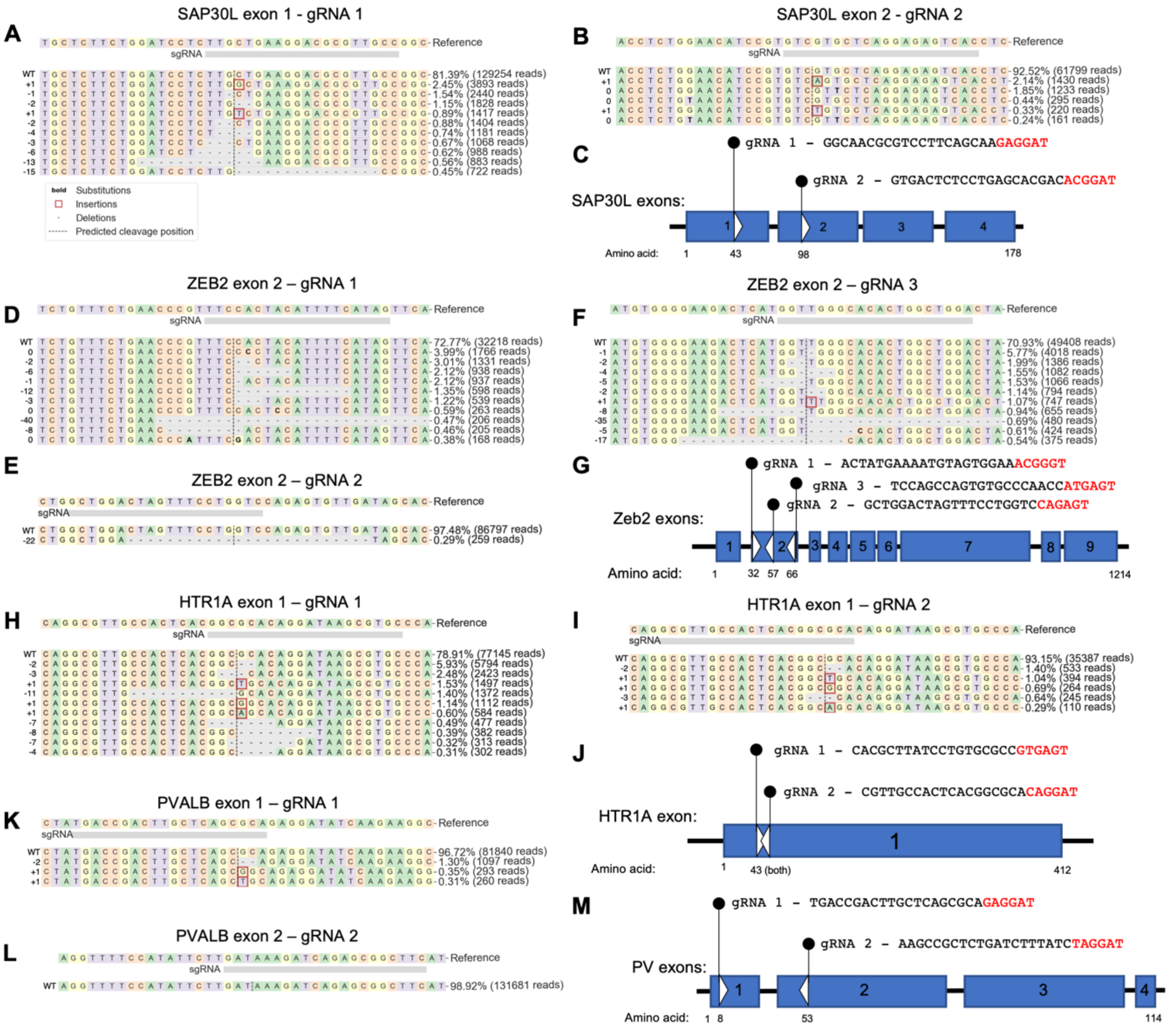

N

gRNA	Targeted indel reads detected (%)	Untargeted indel reads (%)	Predicted off-target location (bTaegut1_v1)	Off-target loci indel reads (%)
SAP30L g1	27,144 (17.09)	12 (0.04)	Chr29:1688218; Chr7:36413910	413(0.75); 60 (0.03)
ZEB2 g1	9,439 (21.32)	195 (0.22)	Chr2:97880309; ChrZ:2782887	23 (0.05); 46 (0.09)
ZEB2 g3	19,448 (27.92)	158 (0.18)	Ch.9: 8776178	44 (0.08)
HTR1A g1	19,440 (19.89)	33 (0.10)	Chr6:14448341; Chr3:52031697	42 (0.07); 82 (0.16)

Supplementary Fig. 6. Top CFS414 indel formations from gene targeting gRNAs.
(A-B) Sequence reads of *SAP30L* loci following targeting by SAP30L (A) exon 1 gRNA 1 and (B) exon 2 gRNA 2. Legend below (A) denotes indel formation type. (C) Diagram of gRNA target sites on the *SAP30L* gene, showing the exon and predicted amino acid codon sequence targeted. (D-F) Sequence reads of *ZEB2* loci following targeting on exon 2 by (D) gRNA 1, (E) gRNA 2, and (F) gRNA 3. (G) Diagram of gRNA target sites on the *ZEB2* gene. (H-I) Sequence reads of *HTR1A* locus following targeting by (H) gRNA 1 and (I) gRNA 2. (J) Diagram of gRNA target sites on the *HTR1A* gene. (K-L) Sequence reads of *HTR1A* locus following targeting by (K) gRNA 1 and (L) gRNA 2. (M) Diagram of gRNA target sites on the *PVALB* gene. All gRNAs show top 10 indel sequences found in more than 0.3% of reads. (N) Table of indel sequence read counts and their relative percent of total reads for the 4 most efficient gRNAs. which shows modified read percentages from targeted and untargeted CFS414 clone 1G10 samples, and gRNA-targeted 1G10 cells at predicted off-target loci.

SUPPLEMENTARY INFORMATION

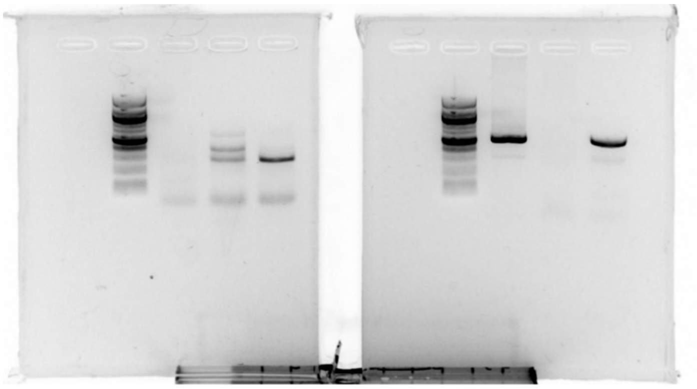

Fig. 1F Unprocessed image of 2% agarose gels, showing PCR DNA amplicon bands. The *CHD* sextyping amplicons are on the left and the *SV40Tt* immortalization construct amplicons are on the right. The samples in each gel are ordered by w612-1 plasmid, wildtype FEF genomic DNA, and CFS414 genomic DNA.
